# Financing and purchasing mechanisms of primary health care in Southeast Asia region: Findings from a scoping review

**DOI:** 10.1016/j.ssmhs.2025.100132

**Published:** 2025-12

**Authors:** Hsu Myat Mon, Aungsumalee Pholpark, Leonard Thomas Sy Lim, Tsolmongerel Tsilaajav, Valeria de Oliveira Cruz, Piya Hanvoravongchai

**Affiliations:** a Faculty of Medicine, Chulalongkorn University, Anantamahidol Building, 1873, Rama 4 Road, Pathumwan, Bangkok 10330, Thailand; b Faculty of Social Sciences and Humanities, Mahidol University, Tambon Salaya, Amphoe Phutthamonthon, Nakhon Pathom 73170, Thailand; c College of Medicine, University of the Philippines Manila, 547 Pedro Gil Street, Ermita, Manila, Philippines; d World Health Organization, South-East Asia Regional Office, World Health House, Indraprastha Estate, Mahatma Gandhi Road, New Delhi 110002, India

**Keywords:** primary health care, health financing, revenue mobilization, pooling, resource allocation, purchasing, Southeast Asia region

## Abstract

**Background:**

Despite commitment to Primary Health Care (PHC), financing has been a persistent challenge in Southeast Asia, with limited discussions. To address this knowledge gap, this study explores three key financing mechanisms: revenue mobilization, pooling, and purchasing across the region.

**Methods:**

A scoping review, with searches in PubMed, Scopus, Embase, and Google Scholar, was conducted. Screening was done via Covidence, with data extracted and analyzed in Excel using a framework analysis.

**Results:**

Of 2521 sources, 171 were included. Limited information specific to PHC was found. Revenue mobilization mainly includes out-of-pocket payment, government funding, social insurance contributions, and a mix of government and external funding. Pooling for PHC financing was seen in Thailand, while other countries showed multiple levels of pooling under general health financing. The prevalent purchasing method for public facilities is line-item budgeting, with salaries as the primary payment method for healthcare providers. Some countries employ performance-linked methods and capitation for provider payments. Significant challenges include inadequate budget allocations, financial flow fragmentation, and poor coordination and low capacity for financial management.

**Conclusion:**

Financing for PHC is found to be insufficient and inefficient, mainly using traditional mechanisms. Cross-country learning and collaboration can support the development of strategic PHC financing mechanisms.

## Highlights

• Varying levels of PHC expenditure were seen among countries in the region.
• Public funding for PHC facilities mainly uses centralized line-item approaches.
• Literature recommends increased public spending and improved financing mechanisms.
• Financial fragmentation and low management capacity were common challenges.
• Most of the information found is focused on general health financing than PHC.

## 1 Introduction

Since the Alma-Ata declaration (2018), primary health care (PHC) has been recognized as one of the most cost-effective strategies to achieve universal health coverage (UHC) (World Health Organization, 2019). Approximately 90 % of essential health services can be delivered through PHC, resulting in its prioritization globally (World Health Organization, 2019, Hanson et al., 2022). Several PHC-focused frameworks and strategies were established to orient health systems towards focusing on PHC, including the Operational Framework for PHC in 2020 (World Health Organization, 2020) and the 2022 approved Regional PHC strategy 2022–2030 (World Health Organization Regional Office for South-East Asia, 2021a).

Nonetheless, overall public health care financing, including PHC, has been a persistent challenge in the South-East Asia Region (SEAR). Government spending on health is ranked as the second lowest among all WHO regions (3.12 % of Gross Domestic Product/GDP in 2022 on average) (World Health Organization Regional Office for South-East Asia, 2021a, World Health Organization, 2025a). The share of Out-Of-Pocket (OOP) expenditure is the highest among all WHO regions (36.35 % of current health expenditure in 2021), albeit with varying levels of OOP expenditure across countries in the region (World Health Organization Regional Office for South-East Asia, 2021a, World Health Organization, 2025a). The region demonstrates considerable variation in gross national income (GNI), current health expenditure (CHE), as well as in the per capita contributions from different funding sources (Table 1).

**Table 1:** Overview of health financing indicators and economic context by country.

Country	Gross National Income (GNI) per capita, Atlas method (current US$) (2024) (World Bank, 2024)	Current Health Expenditure (CHE) as % Gross Domestic Product (GDP) (2022) (World Health Organization, 2025a)	Health Expenditure per Capita in US$(2022) (World Health Organization, 2025a)			
			Domestic General Government Health Expenditure (GGHE-D) per Capita	Domestic Private Health Expenditure (PVT-D) per Capita	Out-of-Pocket Expenditure (OOPS) per Capita	External Health Expenditure (EXT) per Capita
Bangladesh	2820	2	4	46	44	11
Bhutan	3740 (2023)	4	76	56	53	23
Indonesia	4910	3	66	60	42	1
India	2650	3 (2020)	31	47	37	1
Maldives	11650	10	899	219	202	33
Myanmar	1220	5	6	44	38	8
Nepal	1470	7	28	51	49	9
Sri Lanka	3860	4	59	66	21	59
Thailand	7120	5	269	101	34	0
Timor-Leste	1560	14	126	10	10	39

As seen in Table 1, the Maldives has the highest GNI, with the highest per capita expenditure on health in 2022 (US$899 from GGHE-D, US$219 from PVT-D, and US$202 from OOPS). This was followed by Thailand, with US$269 from GGHE-D and US$101 from PVT-D. The lowest health expenditure per capita was seen in Bangladesh and Myanmar, with GGHE-D per capita of US$4 and US$6 respectively. No data was found for the Democratic People’s Republic of Korea (DPR /North Korea).

On the other hand, the SEAR has shown enhanced commitment to PHC, with the health ministers of WHO SEAR countries recently announcing the prioritization of PHC in health budgets at the Seventy-sixth Session of the WHO Regional Committee for SEAR (World Health Organization Regional Office for South-East Asia, 2024). Allocating an optimal proportion of additional resources to PHC is also listed as the first action point of the Delhi Declaration on strengthening PHC. Despite these commitments towards PHC, in most countries of SEAR, health care spending is still strongly focused on secondary and tertiary care (Hanson et al., 2022). The latest available data from six countries in the SEAR shows that, on average, only 32.7 % of total PHC spending was financed by the government in 2018 (World Health Organization, 2025a).

In addition to the inadequate financing, barriers identified in aiming for integrated PHC in the region include 1) operational complexity, 2) bureaucratic rigidities and programmatic silos, 3) conflicts with existing policies and preferences, 4) lack of suitable financing and payment mechanisms, 5) constraints to inter-organizational collaboration, and 6) lack of standardized monitoring systems and built-in evaluations (Asian Development Bank, 2023). Addressing these barriers urgently is necessary while considering the demographic changes of rapid urbanization and the steady shift in the population pyramid marked by the aging process in the region (World Health Organization Regional Office for South-East Asia, 2021a).

Although some information exists on how PHC systems and overall health systems are financed throughout SEAR, the majority are not systematized and gathered within a conceptual framework that allows for the analysis of financing systems and creating appropriate conclusions (Hanson et al., 2022). Systematic and robust financing frameworks are required to analyze and provide recommendations to strengthen PHC financing in the region. In an initial attempt to address this gap, this study reviewed existing literature on the financing mechanisms of PHC in 11 countries in the WHO SEAR: Bangladesh, Bhutan, Democratic People’s Republic of Korea (DPR/North Korea), India, Indonesia, Maldives, Myanmar, Nepal, Sri Lanka, Thailand, and Timor-Leste.

### 1.1 Objective

To systematically examine the three key mechanisms for financing PHC: revenue mobilization, pooling, and resource allocation and purchasing of PHC across 11 countries in the WHO South-East Asia Region, with a specific focus on identifying challenges, best practices, and recommendations discussed in literature.

### 1.2 Research question

What is known from the literature about financing primary health care in the South-East Asia region?

## 2 Methodology

The study employed a scoping review. The research protocol has been registered on the International Platform of Registered Systematic Review and Meta-analysis Protocols (https://inplasy.com/) with the registration number INPLASY202390095.

Operational definitions of PHC and health financing are shown in Box 1, and additional definitions of important terms have been described in Appendix 2. A search strategy was developed by applying the key concepts: PHC, financing, and WHO South-East Asia Region countries. The entire search strategy can be seen in Box 2.

Box 1 **Operational definitions used in the study.**

**Table:** 

Primary health care (PHC)	Primary health care is community-level and first-level health care or the first level of contact of individuals, the family and the community, which includes and integrates healthcare services for health promotion, disease prevention, treatment and management, rehabilitation and palliative care, delivered at both individual and population levels (Hanson et al., 2022, Awofeso, 2004, World Health Organization, 2021a)

Health financing	Health financing is a component of the health system, with key functions of raising revenue, pooling of funds, and purchasing of services to improve the effective service coverage and financial protection (World Health Organization, 2025b).

The existing literature was retrieved through four electronic databases (PubMed, Scopus, Embase, and Google Scholar). The searches in Google Scholar were conducted for each country and SEA region, and the first 30 articles of each search result were exported for review. Additionally, gray literature was searched through reference lists of included articles and hand-searching websites of relevant organizations such as WHO, the World Bank (WB), the Asian Development Bank (ADB), the Asia Pacific Observatory (APO) on Health Systems and Policies, the Alliance for Health Policy and Systems Research, and the Ministries of Health of the eleven countries. Related documents were also identified with the help of country experts.

After identifying the articles, the Covidence software was used for title and abstract screening, conducted by two researchers independently and in a blinded manner (Covidence systematic review software n.d.). Any discrepancies between reviewers were resolved by discussion, and input from a third researcher was sought as needed. Similarly, full-text screening was also done using Covidence by two independent researchers, with the opinion of a third researcher to resolve conflicts (Covidence systematic review software n.d.).

Regarding the inclusion criteria, publications must fulfill the following requirements to be included in the analysis: 1) documents on the financing of PHC of 11 countries in the WHO South-East Asia Region: Bangladesh, Bhutan, DPR Korea/North Korea, India, Indonesia, Maldives, Myanmar, Nepal, Sri Lanka, Thailand, and Timor-Leste, 2) documents written in the English language, 3) documents published between 2000 and the date of search (September 20th 2023). Documents were identified and provided by country experts until December 10th, 2023; thus, the publication period extends until then for grey literature. The collected data was adopted a descriptive approach to examining the current landscape of PHC financing in the South-East Asia Region (SEAR), encompassing narrative accounts, analysis of funding source distribution levels, and contextual explanations. Regarding the exclusion criteria, clinical trials, RCTs, and other economic evaluation studies were excluded from the analysis, and papers were also considered outside of the scope of this review if they researched the impact of PHC financing schemes and economic evaluations such as cost-effectiveness, costing, and modeling of a scheme with no actual implementation of the program.

Following this, data extraction and charting were conducted using a pre-structured framework based on the core functions of PHC financing—namely, revenue mobilization, pooling, and resource allocation and purchasing (World Health Organization Regional Office for South-East Asia, 2021a). In addition, the data extraction included information on bottlenecks, practices with demonstrated good outcomes, as well as recommendations. Following this stage, the results were collated, summarized, and reported using framework analysis and according to the Preferred Reporting Items for Systematic Reviews and Meta-Analyses Extension for Scoping Reviews (PRISMA-ScR) checklist (Tricco et al., 2018).

Box 2 **Search Strategy in PubMed Database.**

**Table:** 

("primary health*"[Title/Abstract] OR "Primary Health Care"[Title/Abstract] OR "PHC"[Title/Abstract] OR "primary care"[Title/Abstract] OR "primary health cent*"[Title/Abstract] OR "primary health service*"[Title/Abstract] OR "primary medical care"[Title/Abstract] OR "first contact of care"[Title/Abstract:∼5] OR "first level of care"[Title/Abstract:∼5] OR "frontline health service*"[Title/Abstract] OR "patient-centered"[Title/Abstract] OR "basic health*"[Title/Abstract] OR "basic health care"[Title/Abstract] OR "basic care"[Title/Abstract] OR "community health"[Title/Abstract] OR "community health care"[Title/Abstract] OR "community care"[Title/Abstract] OR "community health cent*"[Title/Abstract] OR "community health service*"[Title/Abstract] OR "community medical care"[Title/Abstract] OR "comprehensive health*"[Title/Abstract] OR "Comprehensive Health Care"[Title/Abstract] OR "comprehensive care"[Title/Abstract] OR "family practi*"[Title/Abstract] OR "general practi*"[Title/Abstract] OR "Primary Health Care"[MeSH Terms] OR "Community Health Centers"[MeSH Terms] OR "Community Health Services"[MeSH Terms] OR "Comprehensive Health Care"[MeSH Terms]) AND ("health financ*"[Title/Abstract] OR "health system financ*"[Title/Abstract] OR "financ*"[Title/Abstract] OR "revenue rais*"[Title/Abstract] OR "pool*"[Title/Abstract] OR "purchas*"[Title/Abstract] OR "payment*"[Title/Abstract] OR "expenditure*"[Title/Abstract] OR "health budget*"[Title/Abstract] OR "spending*"[Title/Abstract] OR "subsid*"[Title/Abstract] OR "fund*"[Title/Abstract] OR "allocat*"[Title/Abstract] OR "sponsor*"[Title/Abstract] OR "budget*"[Title/Abstract] OR "out of pocket*"[Title/Abstract] OR "out of pocket*"[Title/Abstract] OR "reimburs*"[Title/Abstract] OR "insurance"[Title/Abstract] OR "financing incidence*"[Title/Abstract] OR "Health Care Costs"[MeSH Terms] OR "Cost Allocation"[MeSH Terms] OR "Resource Allocation"[MeSH Terms] OR "Health Expenditures"[MeSH Terms] OR "insurance, health"[MeSH Terms] OR "Financial Management"[MeSH Terms]) AND ("Maldives"[Title/Abstract] OR "Maldive Islands"[Title/Abstract] OR "Maldives"[MeSH Terms] OR "Bangladesh"[Title/Abstract] OR "Bangladesh"[MeSH Terms] OR "Nepal"[Title/Abstract] OR "Nepal"[MeSH Terms] OR "Indonesia"[Title/Abstract] OR "Indonesia"[MeSH Terms] OR "Thailand"[Title/Abstract] OR "Thai"[Title/Abstract] OR "Thailand"[MeSH Terms] OR "India"[Title/Abstract] OR "India"[MeSH Terms] OR "Bhutan"[Title/Abstract] OR "Bhutan"[MeSH Terms] OR "Democratic People's Republic of Korea"[Title/Abstract] OR "DPR Korea"[Title/Abstract] OR "North Korea"[Title/Abstract] OR "Democratic People's Republic of Korea"[MeSH Terms] OR "Sri Lanka"[Title/Abstract] OR "Sri Lanka"[MeSH Terms] OR "East Timor"[Title/Abstract] OR "Timor-Leste"[Title/Abstract] OR "Timor-Leste"[MeSH Terms] OR "Myanmar"[Title/Abstract] OR "Burma"[Title/Abstract] OR "Myanmar"[MeSH Terms] OR "Southeast Asia"[Title/Abstract] OR "south east asia"[Title/Abstract] OR "south east asia"[Title/Abstract] OR "asia, southeastern"[MeSH Terms] OR "asia, southern"[MeSH Terms])

## 3 Results

**Fig. 1:**
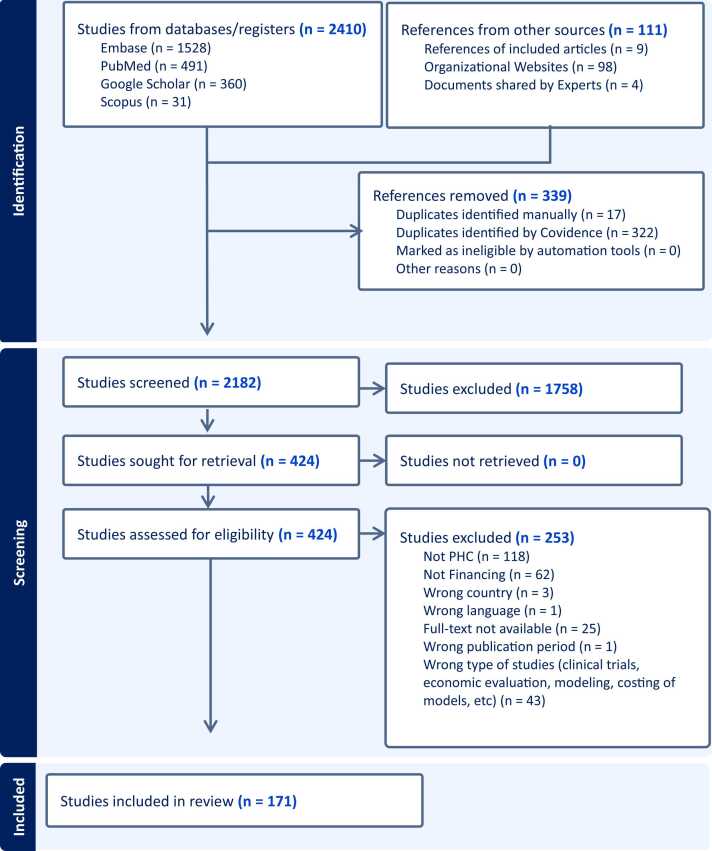
Identification of articles for scoping review (PRISMA Diagram).

A total of 2521 documents were identified. The detailed number of documents from each data source can be seen in Fig. 1. Most of the documents came from Embase and PubMed, with a smaller proportion from Scopus or organizational websites. Altogether, 424 documents were left after de-duplication and title and abstract screening. After full-text screening, 171 documents that focused on the financing of PHC were included in the review. The complete list of documents included in this study is shown in Appendix 1.

There were fewer than five publications per year in the early years of the 21st century. The number started to rise after 2008 with an increasing trend. The count of documents from 2023 is probably lower due to the incomplete year, as indicated by the date of our study. The largest amount of literature was found on India (n = 46), Bangladesh (n = 31), Indonesia (n = 30), and Thailand (n = 29). Fewer publications were found for Nepal (n = 21), Myanmar (n = 17), and Sri Lanka (n = 17). Other countries have less than 10 publications, including Bhutan (n = 7), Maldives (n = 5), Timor-Leste (n = 5), and DPR Korea/North Korea (n = 3). More documents were seen in countries with higher populations, except for DPR Korea/North Korea, with the least number of documents (n = 3) despite having a population larger than four other countries (Sri Lanka, Timor-Leste, Bhutan, and Maldives). Most of the documents were reports, followed by empirical studies (mixed methods, qualitative, and quantitative research), and policy documents and commentaries. A significant number of documents identified were National Health Accounts, with 31 out of 171 documents.

### 3.1 Revenue mobilization

Data on the level of spending on PHC and the sources of PHC expenditure are available only for some countries and specific years (Table 2), while for others, only the information on overall health financing was found. According to the WHO Global Health Expenditure Database, countries in the Southeast Asia (SEA) region exhibit a range of percentages in their primary health care (PHC) expenditure as part of their current health expenditure (CHE). The total PHC expenditure as a percentage of CHE was found to be highest in Bangladesh (67 % in 2020) and Nepal (67 % in 2021). In Thailand, overall PHC expenditure as a percentage of current health expenditure (CHE) was 58 % in 2022; in India, PHC commanded 48 % of CHE in 2019. Among the countries with information on Global Health Expenditure Database, Myanmar and Sri Lanka had the lowest proportion of overall PHC expenditure at 45 % in 2022 and 38 % of CHE in 2018 respectively.

**Table 2:** Per capita expenditure and source-specific allocation of primary health care financing by country.

Country	Primary Health Care (PHC) Expenditure per Capita in US$ (World Health Organization, 2025a)	Primary Health Care (PHC) Expenditure as % Current Health Expenditure (CHE) (World Health Organization, 2025a)	Funding Sources for PHC (World Health Organization, 2025a, World Health Organization Regional Office for South-East Asia, 2021b)		
			Domestic General Government Expenditure on PHC as % PHC	Domestic Private Expenditure on PHC as % PHC	External Expenditure on PHC as % PHC
Bangladesh	34 (2020)	67 (2020)	18 (2020)	76 (2020)	7 (2020)
Bhutan	*No data*	*No data*	79 (2018)	16 (2018)	5 (2018)
Indonesia	*No data*	*No data*	*No data*	*No data*	*No data*
India	29 (2019)	48 (2019)	42 (2019)	57 (2019)	1 (2019)
Maldives	*No data*	*No data*	*No data*	*No data*	*No data*
Myanmar	26 (2022)	45 (2022)	11 (2022)	62 (2022)	26 (2022)
Nepal	44 (2021)	67 (2021)	38 (2021)	47 (2021)	16 (2021)
Sri Lanka	60 (2018)	38 (2018)	26 (2018)	72 (2018)	1 (2018)
Thailand	216 (2022)	58 (2022)	78 (2022)	22 (2022)	0 (2022)
Timor-Leste	*No data*	*No data*	59 (2018)	11 (2018)	30 % (2018)

The distribution of primary health care (PHC) expenditure also revealed differing levels of contribution across various funding sources, including public/government, private, and external entities. In Thailand, most PHC expenditure comes from government funding at 78 % in 2022, and the rest (22 %) was from private expenditure (World Health Organization, 2025a). In Bhutan and Timor-Leste, domestic government revenue was the main contributor of PHC expenditure in 2018 (World Health Organization Regional Office for South-East Asia, 2021b). In Bhutan, in addition to the domestic government revenue (79 %), 16 % of total PHC expenditure was from private financing, with external sources accounting for around 5 % in 2018. Moreover, in Timor-Leste, in 2018, domestic government expenditure (59 %) was followed by external financing at around 30 % and private financing at around 11 %. In Nepal, 38 % of total PHC expenditure was from domestic government expenditure, 47 % was from private expenditure, and 16 % was from external funding in 2021 (World Health Organization, 2025a).

In India, private financing was the primary source of PHC funding at 57 %, followed by government expenditure at 42 % in 2019 (World Health Organization, 2025a, World Health Organization Regional Office for South-East Asia, 2021b). Bangladesh, Sri Lanka and Myanmar follow the same pattern but with even higher percentages of domestic private expenditures at 76 % (2020), 72 % (2018) and 62 % (2022), respectively (World Health Organization, 2025a). The remaining PHC expenditure (26 %) in Sri Lanka mainly came from the government funding in 2018. In addition to government expenditure, Bangladesh had 7 % of external funding (2020), and Myanmar had 26 % of external funding (2022). No PHC-specific expenditure data were found for the Democratic People’s Republic of Korea (DPR Korea/North Korea), Indonesia, and the Maldives in the WHO Global Health Expenditure Database (World Health Organization, 2025a, World Health Organization Regional Office for South-East Asia, 2021b). Furthermore, there is limited information regarding Bhutan and Timor-Leste (World Health Organization, 2025a, World Health Organization Regional Office for South-East Asia, 2021b).

### 3.2 Pooling of funds

From the included literature, minimal information was found on PHC-specific pooling mechanisms in Thailand, and the majority of information pertains to the pooling arrangements for broader health financing in respective countries. In Thailand, the overall health financing has three main pools: the Civil Servants Medical Benefits Scheme (CSMBS) for civil servants and their dependents, the Social Security Scheme (SSS) for formal employees, and the Universal Coverage Scheme (UCS) for Thai citizens not covered by the CSMBS and SSS (Mutiarin et al., 2019). One unique model only found in Thailand is the specific pooling mechanism for prevention and promotion services named the Community Health Fund (CHF). It was established under the Universal Coverage Scheme using a matching fund from the National Health Security Office (NHSO). The local governments that voluntarily participate are required to contribute to the CHF and will receive a per capita budget from the NHSO into this fund (Boonsang and Pongpirul, 2023). A committee is set up with representatives from local government, community leaders, and health workers to manage this fund.

In other countries, only a general health financing function was identified in the literature, where PHC financing would be a part of these pools. In countries with high OOP expenditure (Bangladesh and Myanmar), a large proportion of spending goes through OOP without any pooling mechanism (World Bank, 2021). In Bhutan and the Maldives, pooling functions through one main pool of government revenue mechanisms, where donor funds are combined (World Health Organization Regional Office for South-East Asia, 2021b, World Health Organization, 2021b). The main single pool in Timor-Leste is also composed of government revenue, and only a few donors channel through it (World Health Organization Regional Office for South-East Asia, 2021b). Little information is available on DPR Korea/North Korea, but it is known that their health facilities are funded by centralized health revenues (Noh et al., 2022).

In India, the pooling of funds is managed by state governments (subnational units, similar to a province in other countries) under the country’s decentralized system, with funding divided into flexipools for key programs (e.g., reproductive and child health, communicable and non-communicable diseases) and allocated to State Health Societies via State Treasuries (Selvaraj et al., 2022). Indonesia has two significant pools: central and subnational government budgets for public health services and medical care through their social health insurance called the Jaminan Kesehatan Nasional (JKN) (World Health Organization Regional Office for South-East Asia, 2021b, Bigio et al., 2023). In Nepal, multiple pools are at different levels of government: central, provincial, local governments, and public units (Ministry of Health and Population Nepal, 2023). NGOs and other organizations of national and international origins also pool and manage their funds (Ministry of Health and Population Nepal, 2023). In Sri Lanka, government revenue is collected at the central, provincial, and local levels, where provincial and local governments can also generate their own funds (World Health Organization Regional Office for South-East Asia, 2021b). Similar to Bangladesh and Myanmar, Sri Lanka’s external funds are distributed through the government budget or direct transfers (Ministry of Health Sri Lanka, 2022).

### 3.3 Resource allocation and purchasing mechanisms

Information was limited with respect to resource allocation towards PHC facilities and purchasing mechanisms, for DPR Korea/North Korea, the Maldives, and Timor-Leste. In public PHC facilities, the line-item approach was mainly applied for resource allocation between different levels of governments (World Health Organization Regional Office for South-East Asia, 2021b, Ahmed et al., 2019a). For example, in Bangladesh, the Ministry of Health and Family Welfare allocates budgets directly to upazila health complexes (PHC facilities) using the line-item approach (World Health Organization Regional Office for South-East Asia, 2021b). Performance-based allocations to PHC facilities can be seen in India’s Ayushman Bharat program, Thailand’s Prevention and Promotion program, and Nepal’s Safe Motherhood Program (World Health Organization Regional Office for South-East Asia, 2021b, Selvaraj et al., 2022). For other PHC facilities in Nepal, conditional grants are allocated to the Local Government from the Federal and Provincial Governments through clusters, programs, and budget lines (World Health Organization Regional Office for South-East Asia, 2021b, Selvaraj et al., 2022). In Indonesia, a combination of payment mechanisms is used: capitation for public and private PHC facilities under Badan Penyelenggara Jaminan Sosial Kesehatan, Indonesia’s national health insurance agency; performance-based capitation (Kapitasi Berbasis Komitmen), which adjusts capitation payments based on the performance of PHC providers; and a line-item approach for the salaries and operational costs of public healthcare facilities funded by the Ministry of Health and subnational governments (World Health Organization Regional Office for South-East Asia, 2021b, Mahendradhata et al., 2021). Thailand also uses a mix of approaches. UCS uses both capitation payment and fee schedule, SSS uses capitation-based payments, and CSMBS uses fee-for-service payments (World Health Organization Regional Office for South-East Asia, 2021b, Jongudomsuk et al., 2015). For individual providers, payments were mainly made through salaries (World Health Organization Regional Office for South-East Asia, 2021b, Mutiarin et al., 2019, Rafiei et al., 2021, Perry, 2020, Tangcharoensathien et al., 2016). Fee-for-service was used for private providers in most countries, including Bangladesh, the Maldives, Myanmar, and Nepal (World Health Organization Regional Office for South-East Asia, 2021b, Rajapaksa et al., 2021). Fee-for-service payment was also seen in Indonesia’s screening services (Sambodo et al., 2023, Watabe et al., 2016). For medicines, direct supplies were provided for some countries, while line-item supplies were used for others (World Health Organization Regional Office for South-East Asia, 2021b). Incentives through cash and in-kind items, and travel expenses are provided to volunteers for most PHC services (World Health Organization, 2010).

### 3.4 Practices with good advantages in the region: Insights from published literature

Several practices were identified as beneficial in PHC financing from the literature, with the majority of examples coming from Thailand and Indonesia. These include community-based health fund pooling for prevention and promotion services, a capitation-based payment system, and a purchaser-provider split model where the purchasing of healthcare services is separated from service provision. Strong political commitment and leadership, and the prioritization of cost-effective interventions are also recognized as favorable practices (Table 3).

**Table 3:** Practices with good advantages in the region.

Practices with Good Advantages	Related Countries	References
**Pooling of Funds**		
Community-level pooling of funds for PHC services for rural populations	Thailand, India	(Watabe et al., 2016)
**Resource Allocation and Purchasing**		
Strong prioritization of the small public sector spending on cost-effective and well-targeted interventions	Bangladesh	(Ahmed et al., 2019b, Ahsan et al., 2016)
Uninterrupted medical supplies of quality medical products at PHC centers	Thailand	(Tuangratananon et al., 2021)
Incentives for rural retentions of health staff	Thailand	(Jongudomsuk et al., 2015)
The implementation of public health financing schemes (UCS, SSS for Thailand, JKN for Indonesia) with capitation payment leading to an increase in investment in the primary care system	Thailand, Indonesia	(Hanvoravongchai, 2013, Claramita et al., 2017)
Strategic purchasing with purchaser-provider split model, the option of purchasers (e.g., NHSO for Thailand, BPJS-K for Indonesia) selecting service providers more flexibly	Thailand, Indonesia	(Sambodo et al., 2023, Watabe et al., 2016)
Providing targeted prevention services more efficiently due to capitation combined with performance-based financing	Thailand, Indonesia	(Sambodo et al., 2023, Watabe et al., 2016)
**Governance, Management and Others**		
Sustainable financing, infrastructure and personnel due to strong social welfare policies and leadership from the Ministry of Health (MoH) in the Immunization Program and Epidemiological Surveillance	Sri Lanka	(Abeykoon, 2019)
The establishment of a Regional Public Service Agency (RPSA/BLUD) enhances financial and management flexibility of PHC facilities, while reduce their dependence on the health department	Indonesia	(Shubhan et al., 2020)

### 3.5 Challenges in the region: Insights from published literature

Several challenges were identified in PHC financing in the region, with differences across contexts. Common bottlenecks found in SEA countries are summarized in Table 4. Inadequate budget allocations are a major issue for the overall health sector and PHC, causing high OOP expenditures in some countries. Financial fragmentation and inadequate coordination contribute to inefficiencies and overlaps in PHC services, coupled with a low capacity for financial management in some countries.

**Table 4:** Challenges of PHC financing in SEA countries.

Challenges	Related Countries	References
**Revenue Mobilization**		
High OOP expenditure at the point of health services and/or for purchasing medicines	Bangladesh, Myanmar, Nepal, and India	(Ministry of Health and Population (Nepal), 2023, Health Economics Unit, Ministry of Health and Family Welfare (Bangladesh), 2012, Grundy et al., 2014)
Financial instabilities and insufficiencies in the Urban Primary Health Care Project	Bangladesh	(Ahmad, 2007, Bashar et al., 2022)
**Pooling of Funds**		
Fragmentation of PHC budget and service delivery	Indonesia, Bangladesh	(World Health Organization Regional Office for South-East Asia, 2021b, World Bank, 2020, Evans, 2017)
Resource Allocation and Purchasing		
Inadequate budget allocation to PHC, especially to prevention and promotion services	Indonesia, Thailand, Sri Lanka, Maldives	(Boonsang and Pongpirul, 2023, World Bank, 2020, Ministry of Health, 2018, Ministry of Health, 2019, Perera and Rannan-Eliya, 2017, Withanachchi, 2006)
Disproportionate emphasis on funding specialized care, coupled with low level of government funding for PHC	Sri Lanka, India, Maldives	(Ministry of Health (Sri Lanka), 2022, World Health Organization, 2025 cb; Moosa and Moosa, 2022; Ministry of Health Republic of Maldives, 2019; Afaal, 2002)
Centralized budgeting and planning with unmet local needs	Nepal	(Federal Ministry of Health and Population, 2022)
Underspent budgets and late spending in the financial year due to the slow pace of financial disbursements	Bangladesh, Indonesia, Nepal	(Ahmed et al., 2019a, Ahsan et al., 2016, Evans, 2017, Mahendradhata et al., 2017, Gurung et al., 2016)
Policy incoherence, such as failing to materialize planned projects, lower than expected funding flows, or overly ambitious financial planning	Bangladesh, India, Maldives, and Thailand	(Boonsang and Pongpirul, 2023, Ahmed et al., 2019a, Ahsan et al., 2016, Evans, 2017, Afaal, 2002)
Lack or insufficient remuneration for community health workers	Maldives, Myanmar, Indonesia	(Afaal, 2002, Sommanustweechai et al., 2016)
Private providers’ preference of other payments rather than capitation, resulting in inadequate number of contracted private providers in urban areas	Thailand	(Rokx et al., 2009)
Kapitasi Berbasis Komitmen performance-based capitation by BPJS Kesehatan could not incentivize PHC providers to achieve the targets due to their capacity constraints	Indonesia	(Sambodo et al., 2023)
**Governance, Management and Others**		
Fragmentation and ineffective coordination between agencies in budgeting, pooling, and performance monitoring across line ministries and levels of government	Bangladesh, Indonesia, Thailand	(Boonsang and Pongpirul, 2023, Ahmed et al., 2019a, Ahsan et al., 2016, World Bank, 2020, Evans, 2017)
Inadequate recording, reporting, monitoring and evaluation of expenditures, and lack of accountability	Nepal, Sri Lanka	(Ministry of Health, 2018, Gurung et al., 2016, Patcharanarumol et al., 2018)
Lack of vision and leadership in fund management at local level. Insufficient knowledge and skills for fund management: the absence of a suitable training strategy, especially at the local level	Thailand, Bangladesh	(Ahmed et al., 2019a, Ahsan et al., 2016, Evans, 2017, Patcharanarumol et al., 2018)
Unregulated or loosely structured referral systems, low levels of trust in PHC services, and patients bypassing PHC	Sri Lanka, India, Indonesia	(Rajapaksa et al., 2021, Mahendradhata et al., 2017)
Private providers’ fraudulent in health insurance billing	Thailand	(Islam et al., 2023)
Restrictions or lack of flexibility of facility revenue affecting the quality of services	Indonesia	(Maharania et al., 2022)

### 3.6 Recommendations in the region: Insights from published literature

Based on different challenges and contexts of countries, several recommendations have been identified in the literature (Table 5) (World Health Organization Regional Office for South-East Asia, 2021a). Most documents discussed increasing and prioritizing public spending on health and PHC, focusing on purchasing mechanisms such as capitation and performance-based payments, and strengthening Public Financial Management (PFM) systems to be more efficient and flexible.

**Table 5:** Recommendations for PHC financing in SEA countries.

Recommendations	Related Countries	References
**Revenue Mobilization**		
Prioritizing the national health budget towards integrated PHC and preventive care	Indonesia	(World Bank, 2020, Islam et al., 2023, Claramita et al., 2017)
Collecting tax on harmful products such as tobacco, alcohol, sugary products, and food products with trans-fat and allocating the collected revenue towards PHC services	Bhutan	
User fees collection should be retained to keep PHC free of charge	Bangladesh	(Ahmed et al., 2019a)
Using innovative financing mechanisms, such as tax-based, social and community-based health insurance schemes, or pre-payment schemes that facilitate risk-pooling and risk-sharing functions	Bhutan, Indonesia, India	(World Bank, 2020)
**Resource Allocation and Purchasing**		
Strategic purchasing mechanisms such as mixed provider payment systems considering the full context of respective countries	Indonesia	(World Bank, 2020)
Providing adequate compensation for community health workers	India, Maldives, Myanmar	(Perry, 2020)
Setting higher wage limit and capitation payment rates for Puskesmas	Indonesia	(Supriyana et al., 2019)
Contracting more private PHC providers to address gaps in urban PHC systems	Thailand	(Tangcharoensathien et al., 2016)
Strong PFM system with adequate recording, reporting, and accountability	Nepal, Thailand	(Boonsang and Pongpirul, 2023)
Implementing needs-based local-level budgeting and planning, de-concentration of budget, and rationalization of expenditure control	Nepal	(Ministry of Health and Population MoHP, 2022)
Providing effective training on financial management capacities and fund disbursement processes for relevant officials at all levels	Maldives	(Moosa and Moosa, 2022)

## 4 Discussion

All countries in the SEA region have demonstrated a strong commitment to achieving UHC, with a growing interest in investing in PHC. However, the development of PHC financing and the challenges faced vary significantly across countries due to differences in healthcare systems, financing mechanisms, and levels of economic development.

From this scoping review, an increasing number of academic publications and grey documents contain information on PHC financing in the years after 2015. However, the information was mostly from larger and/or more developed countries such as India, Indonesia, Bangladesh, and Thailand, with minimal information on DPR Korea/North Korea, the Maldives, and Timor-Leste. There was limited information specific to PHC financing, in contrast to the more expansive information available on overall health financing. Countries may face challenges in clearly distinguishing Primary Health Care (PHC) funding from broader health expenditures, which could explain why it is often aggregated in the existing literature. The discussions around PHC financing were mainly on financing mechanisms at different levels, with much less data on resource allocation and provider payments. Countries may benefit from formal and routine measurement of PHC expenditures, comparison and evaluation of expenditures in the region, setting up a regional research agenda, and platforms for cross-country learning. Details on financing mechanisms in Indonesia, along with those in Bangladesh, Maldives, and Nepal, have been discussed in a separate paper as part of this project’s extended work (Pholpark et al., 2024).

Countries in the SEA region have diverse approaches to defining and financing PHC through the provision of levels of care and essential health services packages. On top of this, fragmentation of funding and overlapping of PHC service delivery make PHC financing challenging to measure. This also hinders the ability of the current report to compare and discuss PHC spending across countries. However, PHC financing is seen as relatively underfunded by the governments, a key concern in many low- and middle-income countries. Most countries depend mainly on OOP expenditure for accessing overall health care services, with government revenue being the main source of funding for health care only in some countries. The relative contribution of factors influencing the inadequacy of PHC financing varies across countries, including low overall government revenue, limited prioritization of health within government revenues, and disproportionate allocation of funds away from PHC services.

In terms of pooling, most countries rely on one single pool through government taxation or multiple pooling arrangements at different levels of government and insurance schemes. Using multiple budgeting approaches can lead to inefficiencies and overlaps in health services, increasing fragmentation in how services are delivered and financed. For instance, in Bangladesh, separation can be seen through its dual budgeting system, which separates development and non-development budgets. This results in divided preparation, management, and monitoring processes, hindering cohesive planning and execution within the health sector (Ahmed et al., 2019a). Moreover, key challenges to achieving universal health care and equitable PHC financing include the centralized allocation of resources, which can hinder distributional equity, responsiveness to local needs, and the retention of healthcare professionals in rural and underserved areas. Further research is needed to examine these dynamics and inform targeted strategies that support equitable PHC financing and delivery across the region.

Limited reforms have been observed in the resource allocation and purchasing function of PHC in the SEA region. Public facilities often rely on line-item budgeting, while private sector providers predominantly use fee-for-service payment models. A combination of payment mechanisms was observed only in Indonesia and Thailand, where capitation payments were blended with performance-based payment and fee-for-service/fee schedule (Hanson et al., 2022). The Lancet Global Health Commission on financing primary health care has recommended that the financial arrangements should be people-centred, with a pooled PHC budget allocated and protected equitably based on the people’s needs (Hanson et al., 2022). Transformations should be comprehensive and implemented with various factors such as strategic provider payment mechanisms, capacity building for health-related officials in financial management and procurement processes (Hanson et al., 2022).

Improvements in monitoring, recording, and reporting expenditure for PHC services have been recommended. Community-based primary health care should receive greater attention within facilities and hospitals, as these efforts have been recognized as a priority for accelerating progress toward UHC (Black et al., 2017). Moreover, private providers, community organizations, and non-governmental organizations providing PHC services will need to coordinate effectively in service delivery and in reforming financing mechanisms, ensuring a unified approach towards achieving UHC. Commitments from all associated ministries, through a whole-of-government approach, have been called for in order to allocate more resources and strengthen systems for PHC services (Hanson et al., 2022).

This research project had limited time and resources, posing several limitations to its work. The search strategy focused on popular academic databases, and the papers were restricted to English. Even though we also included grey literature, web searches, and literature from Google Scholar, there is a possibility that existing information on PHC financing written in native non-English languages was missed in our scoping review. Further research and careful tracking of spending on PHC services are necessary to gain a clearer understanding of financing situations in the Southeast Asia region, alongside close monitoring of whether increases and innovations in PHC financing align with growing commitments.

## 5 Conclusion

In all Southeast Asia countries, much of the literature focuses on PHC services or health financing in general, and there is a limited number of studies and reports specifically addressing PHC financing in the region. Moreover, tracking PHC financing remains challenging due to ambiguous definitions, overlapping service delivery across facilities and financing schemes, and the lack of specific measurements of allocations and expenditures. Despite the increasing prioritization of PHC in recent years, several challenges remain, including low budget allocations and fragmentation of financing and service delivery, among others. The type and extent of challenges vary by country, given different health system structures, historical and political contexts, and levels of economic development.

To achieve UHC, health security, and health-related Sustainable Development Goals (SDGs) by 2030, there is an urgent need for improvements in PHC, for which PHC financing is an important function. Policymakers and stakeholders must strengthen all functions of PHC financing, including mobilizing adequate resources, optimizing pooling mechanisms, and ensuring efficient purchasing to achieve equitable and sustainable PHC systems across the region. This must be coupled with a strong capacity for public financial management and monitoring of PHC investments (World Health Organization Regional Office for South-East Asia, 2021a).

## CRediT authorship contribution statement

**Hsu Myat Mon:** Writing – review & editing, Writing – original draft, Visualization, Resources, Project administration, Methodology, Formal analysis, Data curation. **Tsolmongerel Tsilaajav:** Writing – review & editing, Resources, Methodology, Investigation, Formal analysis, Conceptualization. **Valeria de Oliveira Cruz:** Writing – review & editing, Resources, Methodology, Investigation, Formal analysis, Conceptualization. **Aungsumalee Pholpark:** Writing – review & editing, Visualization, Resources, Methodology, Investigation, Formal analysis, Data curation. **Leonard Thomas Sy Lim:** Writing – review & editing, Resources, Formal analysis. **Piya Hanvoravongchai:** Writing – review & editing, Supervision, Resources, Methodology, Investigation, Funding acquisition, Formal analysis, Data curation, Conceptualization.

## Ethical Approval

Ethical approval for the study was received from the Institutional Review Board of the Faculty of Medicine, Chulalongkorn University, Bangkok, Thailand (Certificate of Expedited Review Approval Number 1477/2023).

## Funding

Health Financing and Governance Unit of the Department of Health Systems Development, WHO South-East Asia Regional Office, New Delhi, India.

## Declaration of Competing Interest

The authors declare that they have no known competing financial interests or personal relationships that could have appeared to influence the work reported in this paper.
