# Supplementary material for: Financing and purchasing mechanisms of primary health care in Southeast Asia region: Findings from a scoping review
Source: SSM Health Syst. 2025 Dec;5:100132. doi: 10.1016/j.ssmhs.2025.100132 (PMC12678618; doi:10.1016/j.ssmhs.2025.100132)
Supplement: Supplementary file 1 — Supplementary material [file mmc1.docx]

# Appendix 1. List of Documents (sorted by year of publication)

| Year | Title | Authors | Country |
| --- | --- | --- | --- |
| 2023 | Timor-Leste: A Primary Health Care Case Study In The Context of the COVID-19 Pandemic (1) | Nelson Martins, Caetano Gusmao, Jorge Martins, Odete Viegas | Timor-Leste |
| 2023 | Recovery Of Routine Immunisation: Mapping External Financing Opportunities For Reaching Zero-Dose Children (2) | Sarah Tougher, Nikhil Mandalia and Ulla Kou Griffiths | The Democratic People's Republic of Korea, India, Indonesia, Myanmar |
| 2023 | Contracting The Private Health Sector In Thailand’s Universal Health Coverage (3) | Aniqa Islam Marshall, Woranan Witthayapipopsakul, Somtanuek Chotchoungchatchai, Waritta Wangbanjongkun, Viroj Tangcharoensathien | Thailand |
| 2023 | Development Strategy For The Local Health Security Fund In National Health Security Office Region 10, Ubon Ratchathani, Thailand (4) | Arun Boonsang, Somboon Penpim | Thailand |
| 2023 | Learning From Pandemic Responses: Informing A Resilient And Equitable Health System Recovery In Thailand (5) | Viroj Tangcharoensathien, Jos Vandelaer, Richard Brown, Rapeepong Suphanchaimat, Phiangjai Boonsuk, and Walaiporn Patcharanarumol | Thailand |
| 2023 | Nepal National Health Accounts (2018/19 - 2019/20) (6) | Government of Nepal, Ministry of Health and Population | Nepal |
| 2023 | Myanmar: A Primary Health Care Case Study In The Context of the COVID-19 Pandemic (7) | Ann Meng Hsuan Lin, Nilar Tin | Myanmar |
| 2023 | Effects Of Performance-Based Capitation Payment On The Use Of Public Primary Health Care Services In Indonesia (8) | Novat Pugo Sambodo, Igna Bonfrer, Robert Sparrow, Menno Pradhan, Eddy van Doorslaer | Indonesia |
| 2023 | National Health Accounts, Estimates For India (2019-20) (9) | National Health Systems Resource Centre | India |
| 2023 | Palliative Care Management Committees: A Model Of Collaborative Governance For Primary Health Care (10) | A. Kochuvilayil, S. Rajalakshmi, A. Krishnan, S. M. Vijayanand, V. R. Kutty, T. Iype, R. P. Varma | India |
| 2023 | Disrupting Healthcare? Entrepreneurship As An “Innovative” Financing Mechanism In India's Primary Care Sector (11) | Sandra Barnreuther | India |
| 2023 | Planning And Financing Of RMNCH+A Under National Health Mission: A Case Study Of The Gurugram District Of Haryana State (12) | Ruchi Gaylong, Vijay Kumar Tiwari, T. P. Sherin Raj | India |
| 2023 | A Critical Analysis Of The World's Largest Publicly Funded Health Insurance Program: India's Ayushman Bharat (13) | Rajesh Kamath, Helmut Brand | India |
| 2023 | The Inclusion Of Diagnostics In National Health Insurance Schemes In Cambodia, India, Indonesia, Nepal, Pakistan, Philippines And Viet Nam (14) | Jacob Bigio, Emma Hannay, Madhukar Pai, Bachti Alisjahbana, Rishav Das,Huy Ba Huynh, Uzma Khan, Lalaine Mortera, Thu Anh Nguyen, Muhammad Aamir Safdar, Suvesh Shrestha, A Venkat Raman, Sharat Chandra Verma, Vijayashree Yellappa, Divya Srivastava | Cambodia, India, Indonesia, Nepal, Pakistan,  Philippines and Viet Nam |
| 2023 | Bhutan: A Primary Health Care Case Study In The Context Of The Covid-19 Pandemic (15) | Tashi Tobgay, Sonam Yangchen, Tashi Chozom, Ugyen Tshering, Sonam Wangchuk, Tshokey, Sithar Dorjee | Bhutan |
| 2022 | COVID-19 Health System Response Monitor, Sri Lanka (16) | Lalini C. Rajapaksa, Padmal de Silva, Palitha Abeykoon | Sri Lanka |
| 2022 | National Health Accounts, Sri Lanka (2017 & 2018) (17) | Ministry of Health | Sri Lanka |
| 2022 | Health Sector Budget Analysis: First Five Years Of Federalism (18) | Federal Ministry of Health and Population and British Embassy Kathmandu/Nepal Health Sector Support Programme | Nepal |
| 2022 | Nepal National Health Accounts (2017/18) (19) | Ministry of Health and Population, Government of Nepal | Nepal |
| 2022 | Health Service Readiness, Availability, And Utilization Of Primary Health Care Facilities For Non-Communicable Diseases In Shan state, Myanmar (20) | Win Htike Aung, Nithra Kitreerawutiwong, Orawan Keeratisiroj, Wutthichai Jariya | Myanmar |
| 2022 | PHC Plus Investment Case: Primary Health Care For Universal Health Coverage (21) | Sheena Moosa, Athifa Ibrahim and Mariyam Shafeeq | Maldives |
| 2022 | Sustaining Maternal And Child Health Programs When Donor Funding Ends: A Case Study Of Stakeholder Involvement In Indonesia (22) | Budi Aji, Dian Anandari, Henny Soetikno, and Herman Sumawan | Indonesia |
| 2022 | The National Health Insurance System Of Indonesia And Primary Care Physicians' Job Satisfaction: A Prospective Qualitative Study (23) | Chatila Maharania, Sri Ratna Rahayub, Michael Marxa and Svetla Loukanovac | Indonesia |
| 2022 | Attributes Of Funding Flows And Quality Of Maternal Health Services In A Mixed Provider Payment System: A Cross-Sectional Survey Of 108 Healthcare Providers In Indonesia (24) | Dorit Talia Stein, Mukhammad F. Rakhmadi, Arin Dutta, and Jorge I. Ugaz | Indonesia |
| 2022 | Reiterating The Importance Of Publicly Funded And Provided Primary Healthcare For Non-communicable Diseases: The Case Of India (25) | Sulakshana Nandi | India |
| 2022 | India Health System Review (26) | Sakthivel Selvaraj, Anup K Karan, Swati Srivastava,  Nandita Bhan, Indranil Mukhopadhyay | India |
| 2022 | National Health Accounts, Estimates For India (2018-2019) (27) | National Health Systems Resource Centre | India |
| 2022 | Assessing Community Health Governance For Evidence-Informed Decision-Making: A Cross-Sectional Study Across Nine Districts Of India (28) | Shantanu Sharma, Sucheta Rawat, Faiyaz Akhtar, Rajesh Kumar Singh and Sunil Mehra | India |
| 2022 | Improving Community Health Worker compensation: A Case Study From India Using Quantitative Projection Modeling And Incentive Design Principles (29) | Mokshada Jain, Yael Caplan, Banadakoppa Manjappa Ramesh, Hannah Kemp, Bettina Hammer, Shajy Isac, James Blanchard, Vasanthakumar Namasivayam, Sema K. Sgaiera | India |
| 2022 | Assessing The Time Use And Payments Of Multipurpose Community Health Workers For The Various Roles They Play: A Quantitative Study Of The Mitanin Programme In India (30) | Samir Garg, Mukesh Dewangan, Prabodh Nanda, Krishnendhu C, Ashu Sahu and Lalita Xalxo | India |
| 2022 | Non-Communicable Diseases And Transitioning Health System In The Democratic People’s Republic Of Korea During COVID-19 Lockdown (31) | Jin-Won Noh, Kyoung-Beom Kim, Ha-Eun Jang, Min-Hee Heo, Young-Jin Kim and Jiho Cha | DPR Korea |
| 2022 | Making Doctors Stay: Rethinking Doctor Retention Policy In A Contracted-Out Primary Healthcare Setting In Urban Bangladesh (32) | Farzana BasharID, Rubana Islam, Shaan Muberra Khan, Shahed Hossain, Adel A.S. Sikder, Sifat Shahana Yusuf, Alayne M. Adams | Bangladesh |
| 2021 | Global Health Expenditure Database (176) | WHO | Bangladesh, India, Myanmar, Nepal, Sri Lanka, Thailand |
| 2021 | Perspectives And Understanding Of The Policy Makers And Local Authorities Towards Decentralization And How It Affects Health Financing And Budget Allocation At Municipality Level In Timor-Leste (33) | Joao S Martins, Teresa A Madeira, Jose Dionisio Ximenes, Natalia Pereira, Antonio Ximenes, Manuel Fernandes and Vicente de Paulo Correira | Timor-Leste |
| 2021 | Healthcare System Functions In Iran And Successful Developing Countries Regarding Access To Universal Health Coverage: A Comparative Study (34) | Nahid Hatam, Yegane Partovi, Seyed Reza Najibi, Milad Ahmadi Marzaleh and Seyede Maryam Najibi | Thailand |
| 2021 | Healthcare Providers’ Perspectives On Integrating NCDs Into Primary Healthcare In Thailand: A Mixed Method Study (35) | Titiporn Tuangratananon, Sataporn Julchoo, Mathudara Phaiyarom, Warisa Panichkriangkrai,  Nareerut Pudpong, Walaiporn Patcharanarumol and Viroj Tangcharoensathien | Thailand |
| 2021 | Primary Care Physician Payment Mechanisms Toward Universal Health Coverage: A Study Of Iran And Selected Countries (36) | Narges Rafiei, Soudabeh Vatankhah, and Mohammad Javad Kabir | Thailand |
| 2021 | Sri Lanka: Health System Review (37) | Lalini Rajapaksa, Padmal De Silva, Palitha Abeykoon, Lakshmi Somatunga, Sridharan Sathasivam, Susie Perera, Eshani Fernando, Dileep De Silva, Ashok Perera, Usha Perera, Yasoma Weerasekara, Anuji Gamage, Nalinda Wellappuli, Nimali Widanapathirana, Rangika Fernando, Chatura Wijesundara, Ruwanika Seneviratne, Kusal Weerasinghe | Sri Lanka |
| 2021 | Improved Immunization Access Through Health Systems Strengthening Project For Townships In Myanmar: A Mixed Method Study (38) | Win Yee Mon, Nithat Sirichotiatana, Sukhontha Kongsin, Sukhum Jiamtom, Chardsumon Prutipinyo | Myanmar |
| 2021 | Myanmar - Improving Public Financial Management for Health Services: Challenges and Opportunities for Improving Service Delivery in the Wake of COVID-19 (39) | The World Bank | Myanmar |
| 2021 | COVID-19 Health System Response Monitor: Republic of Indonesia (40) | Yodi Mahendradhata, Ni Luh Putu Eka Putri Andayani, and Tiara Marthias | Indonesia |
| 2021 | The Cost Analysis Of Human Resources Development Stunting Prevention (41) | Hairuddin, Reza Aril Ahri, Nurmiati Muchlis, Masriadi, Nurul Susanti | Indonesia |
| 2021 | Strengthening Primary Health Care As A Means To Achieve Universal Health Coverage: Experience from India (42) | Nima Asgari-Jirhandeh, Tomas Zapata, and Manoj Jhalani | India |
| 2021 | National Health Accounts, Estimates For India (FY 2017-18) (43) | National Health Accounts Technical Secretariat, National Health Systems Resource Centre, Ministry of Health and Family Welfare, Government of India | India |
| 2021 | Ayushman Bharat And Universal Health Coverage In India: Is Our Approach Ethical? (44) | Soham D Bhaduri | India |
| 2021 | National Health Accounts, Bhutan (For Financial Years: 2018-2019 & 2019-2020) (45) | World Health Organization | Bhutan |
| 2021 | Crisis Or Opportunity?: Health Financing In Times Of Uncertainty: Country Profiles From The SEA Region (46) | WHO South-East Asia Region | Bangladesh, Bhutan, India, Indonesia, Maldives, Myanmar, Nepal, Sri Lanka, Thailand, Timor-Leste |
| 2020 | COVID-19 Health System Response Monitor, Thailand (47) | Walaiporn Patcharanaruamol, Angkana Lekagul, Chutima Akaleephan, Kamolphat Markchang, Mathudara Phaiyarom, Nattadhanai Rajatanavin, Nattanicha Pangkariya, Orana Chandrasiri, Orratai Waleewong, Putthipanya Rueangsom, Ratchaporn Congprasert, Repeepong Suphanchaimat, Sataporn Julchoo, Somtanuek Chotchoungchatchai, Titiporn Tuangrattananon, Thinakorn Noree, Warisa Panichkriangkrai, Watinee Kunpuek, Viroj Tangcharoensathien, Anns Issac, Nima Asgari‑Jirhandeh | Thailand |
| 2020 | Myanmar National Health Accounts: Health Expenditure Report (2016-2018) (48) | Thant Sin Htoo, Ye Min Htwe, Maung Maung Htay, Zaw, Phyu Win Thant, Khin Thu Htet, Tun Win Lat, Kaung Myat Oo, Patricia Hernandez Pena | Myanmar |
| 2020 | Spending For Better Results: Indonesia Public Expenditure Review (49) | World Bank | Indonesia |
| 2020 | Optimization Of Primary Health Care To Improve Health Services For Participants Of Social Insurance Administering Agency Of Health In Indonesia (50) | M. Hadi Shubhan, Rr Herini Siti Aisyah, L. Budi Kagramanto, Urip Santoso, Siswanto | Indonesia |
| 2020 | The Dynamics Of Budget Fulfillment Policy In The Implementation Of Minimum Service Standards In The Health Sector At Community Health Centers (Case Study In Sleman And Magelang Regencies, Indonesia) (51) | Sunarto, Mukti, A.G, Pramusinto A. | Indonesia |
| 2020 | Decentralization Implementation Of HIV/AIDS Programs In The Province Of South Sulawesi (52) | Darmawansyah, Muhammad Alwy Arifin, Muh Yusri Abadi, Muhammad Al Fajrin, and Anwar Mallongi | Indonesia |
| 2020 | Universal Health Coverage And Tuberculosis Care In India In The Times Of Covid-19 (53) | Anurag Bhargava, Madhavi Bhargava, Ajay Meher | India |
| 2020 | Factors Influencing The Performance Of Community Health Workers: A Qualitative Study Of Anganwadi Workers From Bihar, India (54) | Aparna John, Nicholas Nisbett, Inka Barnett, Rasmi Avula, Purnima Menon | India |
| 2020 | Health & Wellness Centers To Strengthen Primary Health Care In India: Concept, Progress And Ways Forward (55) | Chandrakant Lahariya | India |
| 2020 | Achieving Health For All: Primary Health Care In Action (56) | Meike Schleiff, David Bishai | Bangladesh, Nepal |
| 2020 | Health For The People: National Community Health Worker Programs From Afghanistan To Zimbabwe (57) | USAID, and Maternal and Child Survival Program | Bangladesh, India, Indonesia, Myanmar, Nepal, Thailand |
| 2020 | Public Expenditure Review 1997-2020 (58) | Health Economics Unit, Health Services Division,  Ministry of Health and Family Welfare | Bangladesh |
| 2019 | Universal Health Coverage And Primary Care, Thailand (59) | Kanitsorn Sumriddetchkajorn, Kenji Shimazaki, Taichi Ono, Tesshu Kusaba, Kotaro Satoc & Naoyuki Kobayashid | Thailand |
| 2019 | Financing Common Goods For Health: Sri Lanka (60) | Palitha Abeykoon | Sri Lanka |
| 2019 | Nepal National Health Accounts (2016/17) (61) | Ministry of Health and Population, Government of Nepal | Nepal |
| 2019 | Maldives: National Health Accounts (2015-2017) (62) | Ministry of Health, Republic of Maldives | Maldives |
| 2019 | Moving Towards Evaluation Sustainable Universal Health Coverage Finance Policy: A Comparison Study Between Indonesia and Thailand (63) | Dyah Mutiarin, Amporn Tamronglak, Suranto, Awang Darumurti, and Sakir | Indonesia and Thailand |
| 2019 | Evaluation Of The Health Policy Implementation Of Indonesian Social Insurance Administration Organization In Primary Health Care Facilities (64) | Supriyana, Edy Susanto, Irmawati, Bernadus Rudy Sunindya, Asep Tata Gunawan, Ismi Rajiani | Indonesia |
| 2019 | An Unintended Consequence Of Provider Payment Reform: The Case Of Capitation Grants In The National Health Insurance Reform Of Indonesia (65) | Si Ying Tan and Jiwei Qian | Indonesia |
| 2019 | The Implementation Of A Chronic Disease Management Program (Prolanis) In Indonesia: A Literature Review (66) | Sesty Rachmawati, Hanni Prihhastuti-Puspitasari, and Elida Zairina | Indonesia |
| 2019 | National Health Accounts, Estimates For India (FY 2016-17) (67) | National Health Systems Resource Centre | India |
| 2019 | India's Health And Wellness Centres: Realizing Universal Health Coverage Through Comprehensive Primary Health Care (68) | Rajani R Ved, Garima Gupta, Shalini Singh | India |
| 2019 | The Challenge Of Additionality: The Impact Of Central Grants For Primary Healthcare On State-Level Spending On Primary Healthcare In India (69) | Diana M. Bowser, Rajesh Jha, Manjiri Bhawalkar, Peter Berman | India |
| 2019 | Bangladesh: Unravelling The 'Good Health at Low Cost' Story (70) | Shakil Ahmed, Tahmina Begum, and Daniel Cotlear | Bangladesh |
| 2019 | Diagnostic Study Of Public Financial Management: To Strengthen Health Financing And Service Delivery In Bangladesh (71) | Shakil Ahmed, Tahmina Begum, and Owen Smith | Bangladesh |
| 2019 | Universal Health Coverage In Bangladesh: Activities, Challenges, And Suggestions (72) | Taufique Joarder, Tahrim Z. Chaudhury, and Ishtiaq Mannan | Bangladesh |
| 2018 | Strategic Purchasing And Health System Efficiency: A Comparison Of Two Financing Schemes In Thailand (73) | Walaiporn Patcharanarumol, Warisa Panichkriangkrai, Angkana Sommanuttaweechai, Kara Hanson, Yaowaluk Wanwong, Viroj Tangcharoensathien | Thailand |
| 2018 | Reorganising Primary Health Care In Sri Lanka: Preserving Our Progress, Preparing Our Future (74) | Ministry of Health, Nutrition and Indigenous Medicine | Sri Lanka |
| 2018 | Sri Lanka: Achieving Pro-Poor Universal Health Coverage Without Health Financing Reforms (75) | Owen Smith | Sri Lanka |
| 2018 | National Health Accounts, Sri Lanka (2014-2016) (76) | Ministry of Health, Nutrition & Indigenous Medicine Sri Lanka | Sri Lanka |
| 2018 | Nepal National Health Accounts (2012/12-2015/16) (77) | Government of Nepal, Ministry of Health and Population | Nepal |
| 2018 | New Forms Of Development: Branding Innovative Ideas And Bidding For Foreign Aid In The Maternal And Child Health Service In Nepal (78) | Radha Adhikari, Pam Smith, Jeevan Raj Sharma and Obindra Bahadur Chand | Nepal |
| 2018 | National Health Accounts, Myanmar (2014-2015) (79) | Ministry of Health and Sports, WHO | Myanmar |
| 2018 | Myanmar Health Financing System Assessment (80) | Hui Sin Teo, Jewelwayne Salcedo Cain | Myanmar |
| 2018 | Implementation Research To Strengthen Health Care Financing Reforms Toward Universal Health Coverage In Indonesia: A Mixed-Methods Approach To Real-World Monitoring (81) | Rena Eichler, Susan Gigli, and Lisa LeRoy | Indonesia |
| 2018 | National Health Accounts, Estimates For India (Financial Year 2015-16) (82) | National Health Systems Resource Centre | India |
| 2018 | Universal Health Coverage In India: Newer Innovations And The Role Of Public Health | Suneela Garg | India |
| 2018 | National Health Accounts, Bhutan (Fiscal Years 2014-15 & 2015-16) | Ministry of Health | Bhutan |
| 2018 | Contracting-Out Urban Primary Health Care In Bangladesh: A Qualitative Exploration Of Implementation Processes And Experience (83) | Rubana Islam, Shahed Hossain, Farzana Bashar, Shaan Muberra Khan, Adel A. S. Sikder, Sifat Shahana Yusuf and Alayne M. Adams | Bangladesh |
| 2017 | Primary Health Care Systems (PRIMASYS): Case Study From Thailand (84) | Thira Woratanarat, Patarawan Woratanarat, Charupa Lekthip | Thailand |
| 2017 | Primary Health Care System (PRIMASYS): Case Study From Sri Lanka (85) | Antoinette Perera, .S.R. Perera | Sri Lanka |
| 2017 | Different Challenges, Different Approaches And Related Expenditures Of Community-Based Tuberculosis Activities By International Non-Governmental Organizations In Myanmar (86) | Wai Wai Han, Saw Saw, Petros Isaakidis, Mohammed Khogali, Anthony Reid, Nguyen Hoa, Ko Ko Zaw and Si Thu Aung | Myanmar |
| 2017 | Revealing The Missing Link: Private Sector Supply-Side Readiness for Primary Maternal Health Services in Indonesia (87) | Wei Aun Yap, Eko Setyo Pambudi, Puti Marzoeki, Jewelwayne Salcedo Cain, and Ajay Tandon | Indonesia |
| 2017 | The Republic Of Indonesia: Health System Review (88) | Yodi Mahendradhata, Laksono Trisnantoro, Shita Listyadewi, Prastuti Soewondo, Tiara Marthias, Pandu Harimurti, and John Prawira | Indonesia |
| 2017 | Primary Health Care Systems (PRIMASYS): Comprehensive Case Sstudy From Indonesia (89) | Mora Claramita, Nur Afrainin Syah, Fitriana Murriya Ekawati, Oryzati Hilman, and Hari Kusnantoc | Indonesia |
| 2017 | National Health Accounts, Estimates For India (2014-15) (90) | National Health Systems Resource Centre | India |
| 2017 | Financial Burden Of Out-Of-Pocket Expenditures For Primary Health Care In Hilly Areas Of Garhwal Region, Uttarakhand, North India (91) | Arti Gupta, B Venkatashiva Reddy, Vandana Semwal, Amit Kumar Singh | India |
| 2017 | Review Of Community Based Healthcare Financing In India (92) | Poornima Tapas, Deepa Pillai | India |
| 2017 | History, International Relations, And Public Health - The Case Of The Democratic People's Republic Of Korea (1953-2015) (93) | John Grundy | DPR Korea |
| 2017 | The Kingdom Of Bhutan: Health System Review (94) | Sangay Thinley, Pandup Tshering, Kinzang Wangmo, Namgay Wangchuk, Tandin Dorji, Tashi Tobgay, Jayendra Sharma | Bhutan |
| 2017 | Primary Health Care Systems (PRIMASYS): Comprehensive Case Study From Bangladesh (95) | Julie Evans, Md. Imtiaz Alam | Bangladesh |
| 2016 | Thailand National Health Accounts (NHA): Studies of Tracking Health Expenditure by Diseases, (2014-2016) (96) | Shaheda Viriyathorn, Vuthiphan Vongmongkol, Anond Kulthanmanusorn, Yaowaluk Wanwong, Nithiwat Saengruang, Woranan Witthayapipopsakul, Walaiporn Patcharanarumol, Viroj Tangcharoensathien | Thailand |
| 2016 | Health System Review: Achievements And Challenges (97) | Viroj Tangcharoensathien, Walaiporn Patcharanarumol, and Warisa Panichkriangkrai | Thailand |
| 2016 | Analysis Of Health Promotion And Prevention Financing Mechanisms In Thailand (98) | Akihito Watabe, Weranuch Wongwatanakul, Thaksaphon Thamarangsi, Phusit Prakongsai, and Motoyuki Yuasa | Thailand |
| 2016 | Diabetes Prevention And Care In The Universal Health Coverage Context: The Example Of Thailand (99) | Walaiporn Patcharanarumol, Warisa Panichkriangkrai, Sangay Wangmo, Jadej Thammatacharee, Masaaki Uechi, Yaowaluk Wanwong | Thailand |
| 2016 | Sri Lanka National Health Accounts (2013) (100) | Health Economics Cell, Ministry of Health, Nutrition & Indigenous Medicine Sri Lanka | Sri Lanka |
| 2016 | Nepal National Health Accounts (2009/10 - 2011/12) (101) | Ministry of Health, Government of Nepal | Nepal |
| 2016 | Governance Challenges In The Nepalese Primary Health Care System: Time To Focus On Greater Community Engagement? (102) | Gagan Gurung, Sarah Derrett, Philip C. Hill and Robin Gauld | Nepal |
| 2016 | Access To Drugs And Out Of Pocket Expenditure In Primary Health Facilities (103) | Arjun Kumar Thapa, Namita Ghimire, Shiva Raj Adhikari | Nepal |
| 2016 | Community Health Worker In Hard-To-Reach Rural Areas Of Myanmar: Filling Primary Health Care Service Gaps (104) | Angkana Sommanustweechai, Weerasak Putthasri, Mya Lay Nwe, Saw Thetlya Aung, Mya Min Theint, Viroj Tangcharoensathien and San Shway Wynn | Myanmar |
| 2016 | Indonesia Health Financing System Assessment: Spend More, Right, And Better (105) | Ajay Tandon, Eko Pambudi, Pandu Harimurti, Emiko Masaki, Ali Subandoro, Puti Marzoeki, Vikram Rajan, Darren W. Dorkin, Amit Chandra, Chantelle Boudreaux , Melissa Chew, and Nugroho Suharno | Indonesia |
| 2016 | Decentralization And Primary Health Care Innovations In Indonesia (106) | Suwatin Miharti, Ronald L. Holzhacker and Rafael Wittek | Indonesia |
| 2016 | National Health Accounts, Estimates For India (2013-14) (107) | National Health Systems Resource Centre | India |
| 2016 | Fifteen Years Of Sector-Wide Approach (SWAp) In Bangladesh Health Sector: An Assessment Of Progress (108) | Karar Zunaid Ahsan, Peter Kim Streatfield, Rashida -E-Ijdi, Gabriela Maria Escudero, Abdul Waheed Khan, and M M Reza | Bangladesh |
| 2015 | The Kingdom Of Thailand: Health System Review (109) | Pongpisut Jongudomsuk, Samrit Srithamrongsawat, Walaiporn Patcharanarumol, Supon Limwattananon,  Supasit Pannarunothai, Patama Vapatanavong, Krisada Sawaengdee, Pinij Fahamnuaypol | Thailand |
| 2015 | The Effect Of Payment And Incentives On Motivation And Focus Of Community Health Workers: Five Case Studies From Low- And Middle-Income Countries (110) | Debra Singh, Joel Negin, Michael Otim, Christopher Garimoi Orach and Robert Cumming | India, Bangladesh, Nepal |
| 2015 | National Health Accounts, Bhutan (2011-12 and 2012-13) (111) |  | Bhutan |
| 2015 | Bangladesh National Health Accounts (1997-2012) (112) | Ministry of Health and Family Welfare, Government of the People's Republic of Bangladesh | Bangladesh |
| 2015 | Bangladesh Health System Review (113) | Syed Masud Ahmed, Bushra Binte Alam, Iqbal Anwar, Tahmina Begum, Rumana Huque, Jahangir AM Khan, Herfina Nababan, Ferdaus Arfina Osman, Aliya Naheed, Krishna Hort | Bangladesh |
| 2014 | National Health Accounts: Myanmar (2012-2013) (114) | Ministry of Health, WHO | Myanmar |
| 2014 | Adapting To Social And Political Transitions – The Influence Of History On Health Policy Formation In The Republic Of The Union Of Myanmar (Burma) (115) | John Grundy, Peter Annear, Shakil Ahmed, Beverley-Ann Biggs | Myanmar |
| 2014 | National Health Accounts, Indonesia (2014) (116) | Center for Health Financing and Insurance, Ministry of  Health Government of Indonesia | Indonesia |
| 2014 | Treatment Seeking And Health Financing In Selected Poor Urban Neighbourhoods In India, Indonesia And Thailand (117) | Jens Seeberg, Supasit Pannarunothai, Retna Siwi Padmawati, Laksono Trisnantoro, Nupur Barua d, Chandrakant S. Pandav | India, Indonesia, Thailand |
| 2014 | The Development Of Mental Health Services Within Primary Care In India: Learning From Oral History (118) | Nadja van Ginneken, Sanjeev Jain, Vikram Patel and Virginia Berridge | India |
| 2014 | Community Health Worker Programs In India: A Rights-Based Review (119) | Kavita Bhatia | India |
| 2013 | Health Financing Reform In Thailand: Toward Universal Coverage Under Fiscal Constraints (120) | Piya Hanvoravongchai | Thailand |
| 2013 | The Cost Of Service Quality Improvements: Tracking The Flow Of Funds In Social Franchise Networks In Myanmar (121) | David Bishai, Amnesty LeFevre, Marc Theuss, Matt Boxshall,John D Hetherington, Min Zaw and Dominic Montagu | Myanmar |
| 2013 | Maldives: National Health Accounts (2011) (122) | Health Economic Unit, Policy Planning Division, Ministry of Health, Maldives | Maldives |
| 2013 | Contracting Urban Primary Healthcare Services In Bangladesh - Effect On Use, Efficiency, Equity And Quality Of Care (123) | Anna Heard, Dhiraj Kumar Nath and Benjamin Loevinsohn | Bangladesh |
| 2012 | The Provincial Health Office As Performance Manager: Change In The Local Healthcare System After Thailand's Universal Coverage Reforms (124) | Siranee Intaranongpai, David Hughes and Songkramchai Leethongdee | Thailand |
| 2012 | Jamkesmas Health Service Fee Waiver: Social Assistance Program And Public Expenditure Review 4 (125) | World Bank | Indonesia |
| 2012 | Health Care Financing Reforms In India (126) | Govinda Marapalli Rao, Mita Choudhury | India |
| 2012 | Implementation Of Rogi Kalyan Samiti (RKS) At Primary Health Centre Durvesh (2009 - 2010) (127) | Saurabh Shrivastava, Prateek Bobhate Shrivastava | India |
| 2012 | Improving Access To Maternity Services: An Overview Of Cash Transfer And Voucher Schemes In South Asia (128) | Kate Jehan, Kristi Sidney, Helen Smith & Ayesha de Costa | Bangladesh, India, Nepal |
| 2012 | Expanding Social Protection For Health Towards Universal Coverage (Health Care Financing Strategy 2012-2032) (129) | Health Economics Unit, Ministry of Health & Family Welfare, Government of the People's Republic of Bangladesh | Bangladesh |
| 2011 | Sri Lanka Health Accounts: National Health Expenditure (1990-2008) (130) | Institute for Health Policy, Colombo, Sri Lanka | Sri Lanka |
| 2011 | The National Free Delivery Policy In Nepal: Early Evidence Of Its Effects On Health Facilities (131) | Sophie Witter, Sunil Khadka, Hom Nath and Suresh Tiwari | Nepal |
| 2011 | Health-Financing Reforms In Southeast Asia: Challenges In Achieving Universal Coverage (132) | Viroj Tangcharoensathien, Walaiporn Patcharanarumol, Por Ir, Syed Mohamed Aljunid, Ali Ghufron Mukti, Kongsap Akkhavong, Eduardo Banzon, Dang Boi Huong, Hasbullah Thabrany, Anne Mill | Indonesia and Thailand |
| 2011 | Financing Health Care For All: Challenges And Opportunities (133) | A K Shiva Kumar, Lincoln C Chen, Mita Choudhury, Shiban Ganju, Vijay Mahajan, Amarjeet Sinha, Abhijit Sen | India |
| 2011 | Predicting Performance In Contracting Of Basic Health Care To NGOs: Experience From Large-Scale Contracting In Uttar Pradesh, India (134) | Anna Heard, Maya Kant Awasthi, Jabir Ali, Neena Shukla and Birger C Forsberg | India |
| 2011 | National Health Accounts, Bhutan (2009-10) (135) | Policy and Planning Division, Ministry of Health, Royal Government of Bhutan | Bhutan |
| 2011 | Bangladesh: Second Urban Primary Health Care Project (136) | Asian Development Bank | Bangladesh |
| 2010 | Thailand Health Financing Review 2010 (137) | Viroj Tangcharoensathien,  Walaiporn Patcharanarumol, Chitpranee Vasavid, Phusit Prakongsai, Pongpisut Jongudomsuk, Samrit Srithamrongswat, Jadej Thammathataree, | Thailand |
| 2010 | Using Economic Levers To Change Behaviour: The Case Of Thailand's Universal Coverage Health Care Reforms (138) | David Hughes, Songkramchai Leethongdee, Sunantha Osiri | Thailand |
| 2010 | Putting People First: A Primary Health Care  Success In Rural India (139) | Carol Vlassoff, Marcel Tanner, Mitchell Weiss, Shobha Rao | India |
| 2010 | Regional Consultation On Innovations In Primary Health Care (140) | World Health Organization, Regional Office for South-East Asia | Bangladesh, Nepal |
| 2010 | Glimpses Of Innovations In Primary Health Care In South-East Asia (141) | World Health Organization | Bangladesh, India, Indonesia, Nepal, Timor Leste |
| 2010 | Economic Evaluation Of Demand-Side Financing (DSF) For Maternal Health In Bangladesh (142) | Health, Nutrition and Population Sector Programme (HNPSP) of the Ministry of Health and Family Welfare | Bangladesh |
| 2010 | Bangladesh National Health Accounts (BNHA-III) 1997-2007 (Part I) (143) | Health Economics Unit | Bangladesh |
| 2010 | Vouchers As Demand Side Financing Instruments For Health Care: A Review Of The Bangladesh Maternal Voucher Scheme (144) | Jean-Olivier Schmidt, Tim Ensor, Atia Hossainc, Salam Khan | Bangladesh |
| 2010 | Bangladesh Health Sector Profile: 2010 (145) | The International Bank for Reconstruction and Development/The World Bank and HLSP/Mott McDonald Ltd | Bangladesh |
| 2009 | Thai National Health Accounts: Sustainable Updates Of 2006 And 2007 And Diversifications (146) | Chitpranee Vasavid, Somjit Janyapong, Pintusorn Hempisut, Kulasake Limpiyakorn, Taweesri Greetong, Pensri Trairat, Nuntachavee Hongnuson, Vorrachaya Latthayaporn, Natsuda Phetchnoon, Boonyisa Augsonsiriopas, Pensri Trameekhun, Saichol Susuk, Jarunee Chanphet, Chaleamrat Ruangwarakom, Prathuangtip Dheravajcharoenchai, Chonlavit Suriyasophaphan, Rangsima Preechachard, Kanjana Tisayaticom, Walaiporn Patcharanarumol, Vuthiphan Vongmongkol, Patarapan Odton, Artidtaya Thiempriwan, Shaheda Viriyathorn, Viroj Tangcharoensathien | Thailand |
| 2009 | Policy Characteristics Facilitating Primary Health Care In Thailand: A Pilot Study In Transitional Country (147) | Krit Pongpirul, Barbara Starfield, Supattra Srivanichakorn and  Supasit Pannarunothai | Thailand |
| 2009 | Health Financing In Indonesia: A Reform Road Map (148) | Claudia Rokx, George Schieber, Pandu Harimurti Ajay Tandon, Aparnaa Somanatha | Indonesia |
| 2009 | Primary Health Care And Public-Private Partnership: An Indian Perspective (149) | Ranabir Pal, Shrayan Pal | India |
| 2009 | Micro-Insurance In Bangladesh: Risk Protection For The Poor? (150) | Wendy J. Werner | Bangladesh |
| 2008 | Good Practices In Health Financing: Lessons From Reforms In Low and Middle-income Countries (151) | The International Bank for Reconstruction and Development / The World Bank | Thailand, Sri Lanka |
| 2008 | An Analytical Report On Female Community Health Volunteers Of Selected Districts Of Nepal (152) | New ERA Study Team | Nepal |
| 2008 | Financing Health Improvements In India (153) | Anil B. Deolalikar, Dean T. Jamison, Prabhat Jha, and Ramanan Laxminarayan | India |
| 2007 | Health Status, Trends, And Issues In Sri Lanka (154) | Rasika S. Jayasekara, Tim Schultz | Sri Lanka |
| 2007 | Social Franchising Of TB Care Through Private GPs In Myanmar: An Assessment Of Treatment Results, Access, Equity And Financial Protection (155) | Knut Lönnroth, Tin Aung, Win Maung, Hans Kluge and Mukund Uplekar | Myanmar |
| 2007 | Provision Of Primary Healthcare Services In Urban Areas Of Bangladesh: The Case Of Urban Primary Health Care Project (156) | Alia Ahmad | Bangladesh |
| 2006 | Timor-Leste Health Sector Review, Meeting Health Challenges And Improving Health Outcomes (157) | World Bank | Timor-Leste |
| 2006 | Healthcare Rationing: A Guide To Policy Directions In Sri Lanka (158) | Nimnath Withanachchi, Yasuo Uchida | Sri Lanka |
| 2006 | Re-activating Primary Health Centres Through Industrial Partnership in Tamilnadu - Is It A Sustainable Model of Partnership? (159) | D. Varatharajan,  D. Wilson Arul Anandan | India |
| 2005 | Health Microinsurance: A Comparative Study Of Three Examples In Bangladesh (160) | Mosleh U Ahmed, Syed Khairul Islam, Md. Abul Quashem, and Nabil Ahmed | Bangladesh |
| 2005 | NGO Contracting Evaluation For The HNP Sector In Bangladesh Evidence And Policy Options (161) | Rafael Cortez | Bangladesh |
| 2003 | Bangladesh National Health Accounts (1999-2001) (162) | Health Economics Unit (HEU), Ministry of Health and Family Welfare, Government of the People's Republic of Bangladesh | Bangladesh |
| 2002 | Local Governance And Community Financing Of Primary Care: Evidence From Nepal (163) | David Bishai, Louis W Niessen, Mohan Shrestha | Nepal |
| 2002 | Financing Healthcare: A Case For Reform In The Maldives (164) | Ahmed Afaal | Maldives |
| 2001 | Sri Lanka National Immunization Program: Financing Assessment (165) | Asian Development Bank | Sri Lanka |
| 2000 | Health Care Systems in Transition III. Sri Lanka, Part I. An Overview of Sri Lanka's Health Care System (166) | Dulitha Fernando | Sri Lanka |
| 2000 | Health Care Systems In Transition III. Bangladesh, Part I. An Overview Of The Health Care System In Bangladesh (167) | J. Patrick Vaughan, Enamul Karim and Kent Buse | Bangladesh |

# Appendix 2: Operational Definitions

| Term | Definition |
| --- | --- |
| Revenue mobilization | The process through which countries raise funds to support the organization and delivery of health services. These revenues are commonly classified as public, private and external. (168) |
| Pooling | The accumulation of prepaid health revenues on behalf of a covered population. (168) |
| Purchasing | Allocation of pooled funds or resources to providers that deliver health care goods and services to the population covered by the defined benefits package. (169) |
| Performance-based allocations | The strategic purchasing mechanism in which health care providers are financially rewarded based on the quality, efficiency, and outcomes of the services they deliver, rather than solely on the volume of services provided. (170) |
| Capitation-based payment | A provider payment mechanism whereby a fixed payment per person is made to providers prospectively for a defined benefits package over a specific period, regardless of what services in the package are ultimately provided. Also called per capita provider payment. (171) |
| Conditional grants | An intragovernmental grant from the national government to the devolved governments with particular conditions or standards. (172) |
| Line-item approach | A strategic purchasing mechanism in which public expenditures are categorized by detailed economic items. (171) |
| Purchaser-provider split model | An arrangement that separates the purchaser, as the agent who decides what will be produced, from the provider, as the agent who delivers the agreed outputs or outcomes. (173) |
| External funding | Direct foreign transfers and foreign transfers distributed by government, encompassing all financial inflows into the national health system from outside the country. (174) |
| GNI per capita, Atlas method (current US$) (2024) | GNI measures the total domestic and foreign value added claimed by residents, at a given period in time, usually a year, expressed in current US dollars using the World Bank Atlas method. (175) |
| Primary Health Care (PHC) Expenditure per Capita | Primary Health Care Expenditure (PHCE) / Population / Exchange rate (NCU to USD) (176) |
| Primary Health Care (PHC) Expenditure as % Current Health Expenditure (CHE) | Primary Health Care Expenditure (PHCE) / Current Health Expenditure (CHE) (176) |
| Domestic General Government Expenditure on PHC as % PHC | Domestic General Government Expenditure on PHC (PHCE_gghe-d) / Primary Health Care Expenditure (PHCE) (176) |
| Domestic Private Expenditure on PHC as % PHC | (Compulsory prepayment (Other, and unspecified, than social insurance contributions) + Voluntary prepayment + Other domestic revenues + Unspecified revenues of health care financing scheme) / Current Health Expenditure (CHE) (176) |
| External Expenditure on PHC as % PHC | External sources of funding on Primary Health Care (PHCE_ext) / Primary Health Care Expenditure (PHCE) (176) |
| Current Health Expenditure (CHE) as % Gross Domestic Product | Current Health Expenditure (CHE) / Gross Domestic Product (GDP) (176) |
| Domestic General Government Health Expenditure (GGHE-D) per Capita | (Transfers from government domestic revenue (allocated to health purposes) + Social insurance contributions) / Population / Exchange rate (National Currency Unit/NCU to USD) (176) |
| Domestic Private Health Expenditure (PVT-D) per Capita | (Compulsory prepayment (Other, and unspecified, than social insurance contributions) + Voluntary prepayment + Other domestic revenues + Unspecified revenues of health care financing schemes) / Population / Exchange rate (NCU to USD) (176) |
| Out-of-Pocket Expenditure (OOPS) per Capita | Household out-of-pocket payment / Population / Exchange rate (NCU to USD) (176) |
| External Health Expenditure (EXT) per Capita | External sources of funding/ Population / Exchange rate (NCU to USD) (176) |

# References

1. Martins N, Martins J, Viegas O. Timor-Leste: a primary health care case study in the context of the COVID-19 pandemic. 2023.
2. Tougher S, Martins N, Greer UK. Recovery of routine immunisation: mapping external financing opportunities for reaching zero-dose children. *Vaccines*. 2023;11(7).
3. Marshall AI, Watt W, Chotchoungchatchai S, Wangbanjongkun W, Tangcharoensathien V. Contracting the private health sector in Thailand’s universal health coverage. 2023.
4. Boonsang A, Pinyopornpanish S. Development strategy for the local health security fund in National Health Security Office Region 10, Ubon Ratchathani, Thailand. *J Popul Ther Clin Pharmacol*. 2023;30(3):e545–58.
5. Tangcharoensathien V, Viroj J, Brown R, Suphanchaimat R, Boonsuk P, Patcharanarumol W. Learning from pandemic responses: informing a resilient and equitable health system recovery in Thailand. *Front Public Health*. 2023;11:1065883.
6. Ministry of Health and Population, Government of Nepal. Nepal National Health Accounts (2018/19 – 2019/20). 2023.
7. Lin AMH, Than N. Myanmar: a primary health care case study in the context of the COVID-19 pandemic. 2023.
8. Sambodo NP, Bonfrer I, Sparrow R, Pradhan M, van Doorslaer E. Effects of performance-based capitation payment on the use of public primary health care services in Indonesia. *Soc Sci Med*. 2023;327.
9. National Health Systems Resource Centre. National Health Accounts: Estimates for India (2019–2020). 2023.
10. Kochuvilayil A, Rajalakshmi S, Krishnan A, Vijayanand SM, Kutty VR, Iype T, et al. Palliative care management committees: a model of collaborative governance for primary health care. *Public Health Action*. 2023;13:12–8.
11. Bärnreuther S. Disrupting healthcare? Entrepreneurship as an “innovative” financing mechanism in India's primary care sector. *Soc Sci Med*. 2023;319.
12. Gaylong R, Thakur VK, Raj TPS. Planning and financing of RMNCH+A under National Health Mission: a case study of the Gurugram District of Haryana State. *Int J Health Plann Manage*. 2023;38(4):951–66.
13. Kamath R, Bhatt H. A critical analysis of the world's largest publicly funded health insurance program: India's Ayushman Bharat. *Int J Prev Med*. 2023;14:20.
14. Bigio J, Holmes E, Pai M, Alisjahbana B, Das R, Huynh HB, et al. The inclusion of diagnostics in national health insurance schemes in Cambodia, India, Indonesia, Nepal, Pakistan, Philippines and Viet Nam. *BMJ Glob Health*. 2023;8(7).
15. Tobgay T, Yangchen S, Chozom T, Tshering U, Wangchuk S, Tshokey, et al. Bhutan: a primary health care case study in the context of the COVID-19 pandemic. 2023.
16. Rajapaksa LC, de Silva P, Abeykoon P. COVID-19 Health System Response Monitor, Sri Lanka. 2022.
17. Ministry of Health. National Health Accounts Sri Lanka (2017 & 2018). 2022.
18. Federal Ministry of Health and Population, British Embassy Kathmandu, Nepal Health Sector Support Programme. Health sector budget analysis: first five years of federalism. 2022.
19. Ministry of Health and Population, Government of Nepal. Nepal National Health Accounts (2017/18). 2022.
20. Aung WH, Kyaw N, Keeratisiroj O, Jariya W. Health service readiness, availability, and utilization of primary health care facilities for non-communicable diseases in Shan State, Myanmar. *Iran J Public Health*. 2022;51(6):1303.
21. Moosa S, Imad A, Shiham MS. PHC Plus investment case: primary health care for universal health coverage. 2022.
22. Aji B, Andadari D, Soetikno H, Sumawan H. Sustaining maternal and child health programs when donor funding ends: a case study of stakeholder involvement in Indonesia. *Int J Health Plann Manage*. 2022;37(4):2049–62.
23. Maharania C, Rakhimov SR, Marx M, Loukanova S. The national health insurance system of Indonesia and primary care physicians' job satisfaction: a prospective qualitative study. *Fam Pract*. 2022;39(1):112–24.
24. Stein DT, Fritzen M, Dutta A, Ugaz JI. Attributes of funding flows and quality of maternal health services in a mixed provider payment system: a cross-sectional survey of 108 healthcare providers in Indonesia. *World Med Health Policy*. 2022;15(2):179–93.
25. Nandi S. Reiterating the importance of publicly funded and provided primary healthcare for non-communicable diseases: the case of India. *Int J Health Policy Manag*. 2022;11(6):847–50.
26. Selvaraj S, Karan A, Srivastava S, Bhan N, Mukhopadhyay I. India health system review. 2022.
27. National Health Systems Resource Centre. National Health Accounts: Estimates for India (2018–2019). 2022.
28. Sharma S, Rajalakshmi S, Akhtar F, Singh RK, Mehra S. Assessing community health governance for evidence-informed decision-making: a cross-sectional study across nine districts of India. *Int J Health Gov*. 2022;27(1):8–20.
29. Jain M, Chatterjee Y, Ramesh BM, Kemp H, Hammer B, Isac S, et al. Improving community health worker compensation: a case study from India using quantitative projection modeling and incentive design principles. *Glob Health Sci Pract*. 2022;10(3).
30. Garg S, Das M, Nanda P, Krishnendhu C, Sahu A, Xalxo L. Assessing the time use and payments of multipurpose community health workers for the various roles they play: a quantitative study of the Mitanin Programme in India. *BMC Health Serv Res*. 2022;22(1):1018.
31. Noh J-W, Kim K-B, Jang H-E, Heo M-H, Kim Y-J, Cha J, editors. Non-communicable diseases and transitioning health system in the Democratic People’s Republic of Korea during COVID-19 lockdown. MDPI; 2022.
32. Bashar F, Islam R, Khan SM, Hossain S, Sikder AA, Yusuf SS, Adams AM. Making doctors stay: Rethinking doctor retention policy in a contracted-out primary healthcare setting in urban Bangladesh. PLoS One. 2022;17(1):e0261720.
33. Martins JS, Martins TAM, Ximenes JD, Pereira N, Ximenes A, Fernandes M, Correira VP. Perspectives and understanding of the policy makers and local authorities towards decentralization and how it affects health financing and budget allocation at municipality level in Timor-Leste. Open J Public Health. 2021;3(2):1022.
34. Hatam N, Pourreza P, Najibi SR, Marzaleh MA, Najibi SM. Healthcare system functions in Iran and successful developing countries regarding access to universal health coverage: a comparative study. Iran Red Crescent Med J. 2021;23(7).
35. Tuangratananon T, Jitthai S, Phaiyarom M, Panichkriangkrai W, Pudpong N, Patcharanarumol W, Tangcharoensathien V. Healthcare providers’ perspectives on integrating NCDs into primary healthcare in Thailand: A mixed-method study. Health Res Policy Syst. 2021;19(1):104.
36. Rafiei N, Kabir MJ. Primary care physician payment mechanisms toward universal health coverage: a study of Iran and selected countries. Int J Health Plann Manage. 2021;37(1):372–86.
37. Rajapaksa L, Abeykoon P, Somatunga L, Sathasivam S, Perera S, Fernando E, et al. Sri Lanka: health system review. Health Syst Transit. 2021.
38. Mon WY, Kongsin S, Jiamtom S, Prutipinyo C. Improved immunization access through Health Systems Strengthening Project for townships in Myanmar: a mixed method study. 2021.
39. The World Bank. Myanmar - improving public financial management for health services: challenges and opportunities for improving service delivery in the wake of COVID-19. 2021.
40. Mahendradhata Y, Pandu NLPE, Marthias T. COVID-19 Health System Response Monitor: Republic of Indonesia. 2021.
41. Hairuddin RA, Muchlis N, Masriadi, Susanti N. The cost analysis of human resources development stunting prevention. Indian J Forensic Med Toxicol. 2021;15(3):3097–104.
42. Asgari-Jirhandeh N, Zahir T, Jhalani M. Strengthening primary health care as a means to achieve universal health coverage: experience from India. J Health Manag. 2021;23(1):20–30.
43. National Health Accounts Technical Secretariat (NHATS), National Health Systems Resource Centre (NHSRC), Ministry of Health and Family Welfare (MoHFW), Government of India. National health accounts, estimates for India (2017-2018). 2021.
44. Bhaduri S. Ayushman Bharat and universal health coverage in India: is our approach ethical? Indian J Med Ethics. 2021;VI(3):1–7.
45. World Health Organization. National health accounts, Bhutan (for financial years: 2018-2019 & 2019-2020). 2021.
46. World Health Organization Regional Office for South-East Asia. Crisis or opportunity? Health financing in times of uncertainty: country profiles from the SEA region. 2021.
47. Patcharanaruamol W, Lekagul A, Akaleephan C, Markchang K, Phaiyarom M, Rajatanavin N, et al. COVID-19 Health System Response Monitor, Thailand. 2020.
48. Htoo TS, Htay ZMM, Thant PW, Htet KT, Lat TW, Oo KM, et al. Myanmar National Health Accounts: health expenditure report 2016–2018. 2020.
49. World Bank. Spending for better results: Indonesia public expenditure review. 2020.
50. Shubhan MH, Apriyanto RHS, Kagramanto LB, Santoso U, Siswanto. Optimization of primary health care to improve health services for participants of Social Insurance Administering Agency of Health in Indonesia. J Crit Rev. 2020;7(13):4498–505.
51. Sunarto M, A.G, Pramusinto A. The dynamics of budget fulfillment policy in the implementation of minimum service standards in the health sector at community health centers (case study in Sleman and Magelang regencies, Indonesia). Indian J Public Health Res Dev. 2020;11(3):1172–7.
52. Arifin MA, Yusri MYA, Fajrin MA, Mallongi A. Decentralization implementation of HIV/AIDS programs in the province of South Sulawesi. Medico Legal Update. 2020;20(4):1126–30.
53. Bhargava A, Meher B. Universal health coverage and tuberculosis care in India in the times of COVID-19. Natl Med J India. 2020;33(5):298–301.
54. John A, Nair N, Barnett I, Avula R, Menon P. Factors influencing the performance of community health workers: a qualitative study of Anganwadi workers from Bihar, India. PLoS One. 2020;15(11).
55. Lahariya C. Health & wellness centers to strengthen primary health care in India: concept, progress and ways forward. Indian J Pediatr. 2020;87(11):916–29.
56. Schleiff M, Bzdak D. Achieving health for all: primary health care in action. Baltimore: JHU Press; 2020.
57. USAID, Management Sciences for Health, Community of Practice on Scaling Up CHW Programs. Health for the people: national community health worker programs from Afghanistan to Zimbabwe. 2020:257–72.
58. Health Economics Unit, Health Systems Division, Ministry of Health and Family Welfare. Public expenditure review 1997–2020. 2020.
59. Sumriddetchkajorn K, Suphanchaimat R, Ono T, Kusaba T, Sato K, Kobayashi N. Universal health coverage and primary care, Thailand. Bull World Health Organ. 2019;97(6):415–22.
60. Abeykoon P. Financing common goods for health: Sri Lanka. Health Syst Reform. 2019;5(4):397–401.
61. Ministry of Health and Population, Government of Nepal. Nepal National Health Accounts (2016/17). 2019.
62. Ministry of Health, Republic of Maldives. Maldives: National Health Accounts (2015–2017). 2019.
63. Mutiarin D, Taufik A, Suranto, Darumurti A, Sakir. Moving towards evaluation sustainable universal health coverage finance policy: a comparison study between Indonesia and Thailand. Prosiding ICOGISS. 2019:669–89.
64. Supriyana E, Irmawati, Sunindya BR, Gunawan AT, Rajiani I. Evaluation of the health policy implementation of Indonesian Social Insurance Administration Organization in primary health care facilities. Executive Editor. 2019;10(1):581.
65. Tan SY, Qian J. An unintended consequence of provider payment reform: the case of capitation grants in the National Health Insurance reform of Indonesia. Int J Health Plann Manage. 2019;34(4):e1688–710.
66. Rachmawati S, Handayani HP, Zairina E. The implementation of a chronic disease management program (Prolanis) in Indonesia: a literature review. J Basic Clin Physiol Pharmacol. 2019;30(6).
67. National Health Systems Resource Centre. National Health Accounts, Estimates for India (FY 2016–17). 2019.
68. Ved R, Gupta G, Singh S. India's health and wellness centres: realizing universal health coverage through comprehensive primary health care. WHO South-East Asia J Public Health. 2019;8(1):18–20.
69. Bowser DM, Joshi R, Bhawalkar M, Berman P. The challenge of additionality: the impact of central grants for primary healthcare on state-level spending on primary healthcare in India. Int J Health Policy Manag. 2019;8(6):329–36.
70. Ahmed S, Barkat A, Cotlear D. Bangladesh: unravelling the ‘good health at low cost’ story. 2019.
71. Ahmed S, Barkat A, Smith O. Diagnostic study of public financial management: to strengthen health financing and service delivery in Bangladesh. 2019.
72. Joarder T, Zinia TZ, Mannan I. Universal health coverage in Bangladesh: activities, challenges, and suggestions. Adv Public Health. 2019;2019.
73. Patcharanarumol W, Sommanuttaweechai A, Hanson K, Wanwong Y, Tangcharoensathien V. Strategic purchasing and health system efficiency: a comparison of two financing schemes in Thailand. PLoS One. 2018;13(4).
74. Ministry of Health, Nutrition and Indigenous Medicine. Reorganising primary health care in Sri Lanka: preserving our progress, preparing our future. 2018.
75. Smith O. Sri Lanka: achieving pro-poor universal health coverage without health financing reforms. Universal Health Coverage Study Series No. 38. 2018.
76. Ministry of Health, Nutrition and Indigenous Medicine, Sri Lanka. National Health Accounts, Sri Lanka (2014–2016). 2018.
77. Government of Nepal, Ministry of Health and Population. Nepal National Health Accounts (2012/13–2015/16). 2018.
78. Adhikari R, Sharma JR, Chand OB. New forms of development: branding innovative ideas and bidding for foreign aid in the maternal and child health service in Nepal. Glob Health. 2018;14(1).
79. Ministry of Health and Sports. National Health Accounts, Myanmar (2014–2015). 2018.
80. Teo HS, Chowdhury S. Myanmar health financing system assessment. 2018.
81. Eichler R, Shaw G, LeRoy L. Implementation research to strengthen health care financing reforms toward universal health coverage in Indonesia: a mixed-methods approach to real-world monitoring. Glob Health Sci Pract. 2018;6(4):747–53.
82. National Health Systems Resource Centre. National Health Accounts, Estimates for India (Financial Year 2015–16). 2018.
83. Islam R, Hossain S, Bashar F, Khan SM, Sikder AAS, Yusuf SS, et al. Contracting-out urban primary health care in Bangladesh: a qualitative exploration of implementation processes and experience. Int J Equity Health. 2018;17:1–16.
84. Woratanarat T, Wibulpolprasert S, Lekthip C. Primary health care systems (PRIMASYS): case study from Thailand. 2017.
85. Perera A, Rasanayagam S. Primary health care systems (PRIMASYS): case study from Sri Lanka. 2017.
86. Han WW, Shewade HD, Isaakidis P, Khogali M, Reid A, Hoa N, et al. Different challenges, different approaches and related expenditures of community-based tuberculosis activities by international non-governmental organizations in Myanmar. Infect Dis Poverty. 2017;6(1).
87. Yap WA, Putri S, Marzoeki P, Cain JS, Tandon A. Revealing the missing link: private sector supply-side readiness for primary maternal health services in Indonesia. 2017.
88. Mahendradhata Y, Trisnantoro L, Listyadewi S, Soewondo P, Marthias T, Harimurti P, et al. The Republic of Indonesia: health system review. 2017.
89. Claramita M, Setyaningrum NA, Ekawati FM, Hilman O, Kusnanto H. Primary health care systems (PRIMASYS): comprehensive case study from Indonesia. Geneva: World Health Organization; 2017.
90. National Health Systems Resource Centre. National Health Accounts, Estimates for India (2014–15). 2017.
91. Gupta A, Rawat BV, Semwal V, Singh AK. Financial burden of out-of-pocket expenditures for primary health care in hilly areas of Garhwal region, Uttarakhand, North India. J Clin Diagn Res. 2017;11(4):LC08–11.
92. Tapas P, Dash D. Review of community based healthcare financing in India. Indian J Public Health Res Dev. 2017;8(4):399–406.
93. Grundy J. History, international relations, and public health: the case of the Democratic People's Republic of Korea (1953–2015). Korea's Economy. 2017;31.
94. Thinley S, Tandin P, Wangmo K, Wangchuk N, Dorji T, Tobgay T, et al. The Kingdom of Bhutan: health system review. Health Syst Transit. 2017;7(2).
95. Evans J, Ahmed MIA. Primary Health Care Systems (PRIMASYS): comprehensive case study from Bangladesh. Geneva: World Health Organization; 2017.
96. Viriyathorn S, Vongmongkol V, Kulthanmanusorn A, Wanwong Y, Saengruang N, Witthayapipopsakul W, et al. Thailand National Health Accounts (NHA): studies of tracking health expenditure by diseases (2014–2016). 2016.
97. Tangcharoensathien V, Patcharanarumol W, Panichkriangkrai W. Health system review: achievements and challenges. 2016.
98. Watabe A, Widyastuti W, Thamarangsi T, Prakongsai P, Yuasa M. Analysis of health promotion and prevention financing mechanisms in Thailand. Health Promot Int. 2016;32(4):702–10.
99. Patcharanarumol W, Wangmo S, Thammatacharee J, Uechi M, Wanwong Y. Diabetes prevention and care in the universal health coverage context: the example of Thailand. WHO South-East Asia J Public Health. 2016;5(1):27–33.
100. Health Economics Cell, Ministry of Health, Nutrition & Indigenous Medicine, Sri Lanka. Sri Lanka National Health Accounts (2013). 2016.
101. Ministry of Health, Government of Nepal. Nepal National Health Accounts (2009/10–2011/12). 2016.
102. Gurung G, Dixit S, Hill PC, Gauld R. Governance challenges in the Nepalese primary health care system: time to focus on greater community engagement? Int J Health Plann Manage. 2016;31(2):167–74.
103. Thapa AK, Ghimire N, Adhikari SR. Access to drugs and out-of-pocket expenditure in primary health facilities. J Nepal Health Res Counc. 2016;14(34):139–42.
104. Sommanustweechai A, Patcharanarumol W, Nwe ML, Aung ST, Theint MM, Tun SS, et al. Community health worker in hard-to-reach rural areas of Myanmar: filling primary health care service gaps. Hum Resour Health. 2016;14(1):64.
105. Tandon A, Evans P, Harimurti P, Masaki E, Subandoro A, Marzoeki P, et al. Indonesia health financing system assessment: spend more, right, and better. 2016.
106. Miharti S, Hardiyanti RL, Wahyudi RW. Decentralization and primary health care innovations in Indonesia. In: Decentralization and Governance in Indonesia. 2016:53–78.
107. National Health Systems Resource Centre. National Health Accounts, Estimates for India (2013–14). 2016.
108. Ahsan KZ, Shafiq PK, Ijdi R-E, Escudero GM, Khan AW, Reza MM. Fifteen years of Sector-Wide Approach (SWAp) in Bangladesh health sector: an assessment of progress. 2016.
109. Jongudomsuk P, Srisasalux S, Patcharanarumol W, Tangcharoensathien V, Sawaengdee K, Pannarunothai S, et al. The Kingdom of Thailand: health system review. 2015.
110. Singh D, Negin J, Otim M, Orach CG, Cumming R. The effect of payment and incentives on motivation and focus of community health workers: five case studies from low- and middle-income countries. Hum Resour Health. 2015;13:58.
111. World Health Organization. National Health Accounts, Bhutan (2011–12 and 2012–13). 2015.
112. Ministry of Health and Family Welfare, Government of the People's Republic of Bangladesh. Bangladesh National Health Accounts (1997–2012). 2015.
113. Ahmed SM, Bhuiya B, Anwar I, Begum T, Huque R, Khan JAM, et al. Bangladesh health system review. 2015.
114. Ministry of Health and WHO. National Health Accounts: Myanmar (2012–2013). 2014.
115. Grundy J, Annear P, Ahmed S, Biggs B. Adapting to social and political transitions – the influence of history on health policy formation in the Republic of the Union of Myanmar (Burma). Soc Sci Med. 2014;107:179–88.
116. Center for Health Financing and Insurance, Ministry of Health, Government of Indonesia. National Health Accounts, Indonesia (2014). 2014.
117. Seeberg J, Pannarunothai S, Padmawati RS, Trisnantoro L, Barua N, Pandav CS. Treatment seeking and health financing in selected poor urban neighbourhoods in India, Indonesia and Thailand. Soc Sci Med. 2014;102:49–57.
118. van Ginneken N, Jain S, Patel V, Berridge V. The development of mental health services within primary care in India: learning from oral history. Int J Ment Health Syst. 2014;8(1).
119. Bhatia K. Community health worker programs in India: a rights-based review. Perspect Public Health. 2014;134(5):276–82.
120. Hanvoravongchai P. Health financing reform in Thailand: toward universal coverage under fiscal constraints. 2013.
121. Bishai D, Lonnroth A, Theuss M, Boxshall M, Hetherington JD, Zaw M, et al. The cost of service quality improvements: tracking the flow of funds in social franchise networks in Myanmar. Cost Eff Resour Alloc. 2013;11(1).
122. Health Economic Unit, Planning and Policy Division, Ministry of Health, Maldives. Maldives: National Health Accounts (2011). New Delhi: WHO Regional Office for South-East Asia; 2013.
123. Heard A, Nath DK, Loevinsohn B. Contracting urban primary healthcare services in Bangladesh – effect on use, efficiency, equity and quality of care. Trop Med Int Health. 2013;18(7):861–70.
124. Intaranongpai S, Hanvoravongchai P, Lekagul S. The provincial health office as performance manager: change in the local healthcare system after Thailand's universal coverage reforms. Int J Health Plann Manage. 2012;27(4):308–26.
125. World Bank. Jamkesmas Health Service Fee Waiver: Social Assistance Program and Public Expenditure Review 4. 2012.
126. Rao GM, Choudhury M. Health care financing reforms in India. New Delhi: National Institute of Public Finance and Policy; 2012.
127. Shrivastava S, Bhaisare P. Implementation of Rogi Kalyan Samiti (RKS) at Primary Health Centre Durvesh (2009–2010). TAF Prev Med Bull. 2012;11(3):307–14.
128. Jehan K, Smith H, de Costa A. Improving access to maternity services: an overview of cash transfer and voucher schemes in South Asia. Reprod Health Matters. 2012;20(39):142-54.
129. Health Economics Unit, Ministry of Health and Family Welfare, Government of the People's Republic of Bangladesh. Expanding social protection for health towards universal coverage (Health Care Financing Strategy 2012-2032). 2012.
130. Institute for Health Policy, Sri Lanka. Sri Lanka Health Accounts: National Health Expenditure (1990-2008). Health Accounts Unit, Institute for Health Policy; 2011. 84 p.
131. Witter S, Khadka S, Nath H, Tiwari S. The national free delivery policy in Nepal: early evidence of its effects on health facilities. Health Policy Plan. 2011;26 Suppl 2:ii84-91.
132. Tangcharoensathien V, Por Ir, Aljunid SM, Mukti AG, Akkhavong K, Banzon E, et al. Health-financing reforms in Southeast Asia: challenges in achieving universal coverage. Lancet. 2011;377(9768):863-73.
133. Kumar AKS, Choudhury M, Ganju S, Mahajan V, Sinha A, Sen A. Financing health care for all: challenges and opportunities. Lancet. 2011;377(9766):668-79.
134. Heard A, Kraushaar M, Ali J, Shukla N, Forsberg BC. Predicting performance in contracting of basic health care to NGOs: experience from large-scale contracting in Uttar Pradesh, India. Health Policy Plan. 2011;26 Suppl 1:i13-9.
135. Policy and Planning Division, Ministry of Health, Royal Government of Bhutan. National Health Accounts, Bhutan (2009-10). 2011.
136. Asian Development Bank. Bangladesh: Second Urban Primary Health Care Project. 2011.
137. Tangcharoensathien V, Patcharanarumol W, Vasavid C, Prakongsai P, Jongudomsuk P, Srithamrongswat S, et al. Thailand health financing review 2010. Available from: SSRN 1623260. 2010.
138. Hughes D, Osiri S. Using economic levers to change behaviour: the case of Thailand's universal coverage health care reforms. Soc Sci Med. 2010;70(3):447-54.
139. Vlassoff C, Mitchell M, Weiss M, Rao S. Putting people first: a primary health care success in rural India. Indian J Community Med. 2010;35(2):326.
140. World Health Organization Regional Office for South-East Asia. Regional consultation on innovations in primary health care. WHO SEARO; 2010.
141. World Health Organization. Glimpses of innovations in primary health care in South-East Asia. WHO Regional Office for South-East Asia; 2010.
142. Health N, Planning S, Ministry of Health and Family Welfare. Economic evaluation of demand-side financing (DSF) for maternal health in Bangladesh. 2010.
143. Health Economics Unit. Bangladesh National Health Accounts (BNHA-III) 1997-2007 (Part I). 2010.
144. Schmidt JO, El Arifeen S, Hossain A, Khan S. Vouchers as demand side financing instruments for health care: a review of the Bangladesh maternal voucher scheme. Health Policy. 2010;96(2):98-107.
145. The International Bank for Reconstruction and Development/The World Bank and HLSP/Mott McDonald Ltd. Bangladesh health sector profile. 2010.
146. Vasavid C, Hempisut P, Limpiyakorn K, Greetong T, Trairat P, Hongnuson N, et al. Thai National Health Accounts: sustainable updates of 2006 and 2007 and diversifications. 2009.
147. Pongpirul K, Srivanichakorn S, Pannarunothai S. Policy characteristics facilitating primary health care in Thailand: a pilot study in transitional country. Int J Equity Health. 2009;8.
148. Rokx C, Soewondo P, Harimurti P, Tandon A. Health financing in Indonesia: a reform road map. 2009.
149. Pal R. Primary health care and public-private partnership: an Indian perspective. Ann Trop Med Public Health. 2009;2(2):46-52.
150. Werner WJ. Micro-insurance in Bangladesh: risk protection for the poor? J Health Popul Nutr. 2009;27(4):563-73.
151. The International Bank for Reconstruction and Development / The World Bank. Good practices in health financing: lessons from reforms in low and middle-income countries. 2008.
152. New ERA Study Team. An analytical report on female community health volunteers of selected districts of Nepal. 2008.
153. Deolalikar A, Jha P, Laxminarayan R. Financing health improvements in India. Health Aff (Millwood). 2008;27(4):978-90.
154. Jayasekara RS. Health status, trends, and issues in Sri Lanka. Nurs Health Sci. 2007;9(3):228-33.
155. Lönnroth K, Thu A, Maung W, Kluge H, Uplekar M. Social franchising of TB care through private GPs in Myanmar: an assessment of treatment results, access, equity and financial protection. Health Policy Plan. 2007;22(3):156-66.
156. Ahmad A. Provision of primary healthcare services in urban areas of Bangladesh: the case of Urban Primary Health Care Project. Working paper; 2007.
157. World Bank. Meeting health challenges and improving health outcomes report, Timor-Leste health sector review. 2006.
158. Withanachchi N, Uluwaduge Y. Healthcare rationing: a guide to policy directions in Sri Lanka. Health Policy. 2006;78(1):17-25.
159. Varatharajan D, Anandan DW. Re-activating primary health centres through industrial partnership in Tamil Nadu—is it a sustainable model of partnership? 2006.
160. Ahmed MU, Islam SK, Quashem MA, Ahmed N. Health microinsurance: a comparative study of three examples in Bangladesh. 2005.
161. Cortez R. NGO contracting evaluation for the HNP sector in Bangladesh: evidence and policy options. 2005.
162. Health Economics Unit, Ministry of Health and Family Welfare, Government of the People's Republic of Bangladesh. Bangladesh National Health Accounts (1999-2001). 2003.
163. Bishai D, Shrestha M, Laxminarayan R. Local governance and community financing of primary care: evidence from Nepal. Health Policy Plan. 2002;17(2):202-6.
164. Afaal A. Financing healthcare: a case for reform in the Maldives. Manchester: University of Manchester; 2002.
165. Asian Development Bank. Sri Lanka National Immunization Program: financing assessment. 2001. 47 p.
166. Fernando D. Health care systems in transition III. Sri Lanka, part I. An overview of Sri Lanka’s health care system. J Public Health Med. 2000;22(1):14-20.
167. Vaughan JP, Khan KA, Karim E. Health care systems in transition III. Bangladesh, part I. An overview of the health care system in Bangladesh. J Public Health Med. 2000;22(1):5-9.
168. World Health Organization. Rehabilitation in health financing: opportunities on the way to universal health coverage. Geneva: WHO; 2023. Licence: CC BY-NC-SA 3.0 IGO.
169. World Health Organization. Health systems financing: the path to universal coverage. World Health Report 2010. Geneva: WHO; 2010.
170. McIntyre D, Kutzin J. Health financing country diagnostic: a foundation for national strategy development. Geneva: WHO; 2016 (Health Financing Guidance No. 1). Licence: CC BY-NC-SA 3.0 IGO.
171. World Health Organization. Health financing [Internet]. Geneva: WHO; [cited 2025 Jul 8]. Available from: https://www.who.int/health-topics/health-financing#tab=tab_1
172. Chen A, Mulaki A, Williamson T. Incentivizing performance: conditional grants in Kenya's health system. Washington, DC: Futures Group, Health Policy Project; 2014.
173. Ryan NF, Parker RL, Brown KA. Purchaser-provider split in a traditional public service environment: three case studies of managing change. Public Policy Adm J. 2000;9:206-21.
174. World Health Organization. External health expenditure (EXT) as percentage of current health expenditure (CHE) (%). WHO Global Health Observatory indicator metadata registry [Internet]. [cited 2025 Jul 8]. Available from: https://www.who.int/data/gho/indicator-metadata-registry/imr-details/4955
175. World Health Organization. Indicator metadata registry: civil registration coverage of births (%) [Indicator 1143]. Global Health Observatory [Internet]. 2025 [cited 2025 Jul 8]. Available from: https://www.who.int/data/gho/indicator-metadata-registry/imr-details/1143
176. World Health Organization. Global Health Expenditure Database: view data by indicator [Internet]. [cited 2025 Jul 7]. Available from: https://apps.who.int/nha/database/ViewData/Indicators/en
